# Supplementary material for: Association between low levels of HIV-1 DNA and HLA class I molecules in chronic HIV-1 infection
Source: PLoS One. 2022 Mar 15;17(3):e0265348. doi: 10.1371/journal.pone.0265348 (PMC8923435; doi:10.1371/journal.pone.0265348)
Supplement: S1 File — (DOCX) [file pone.0265348.s001.docx]

***S*YNOPSIS**

| **Protocol Number** *(to be completed after approval)***:** |
| --- |
| **Protocol Title: Monitored Antiretroviral Pause in Chronic HIV-Infected Subjects with Long-Lasting Suppressed Viremia (APACHE Study)** |
| **Country: *Italy*** |
| **Investigator:**  Prof. Adriano Lazzarin |
| **Study type:**  **X Interventional** 🞎 Within-Label 🞎 Off-Label  🞎 Non-Interventional  **X Single Investigator** 🞎 Network of Investigators |
| **Study Phase:**  🞎 Phase I 🞎 Phase II 🞎 Phase III 🞎 Phase IV |
| **Study Design**  **X Open-Label** 🞎 Randomized 🞎 Double-Blind 🞎 Single-Blind  🞎 Crossover 🞎 Parallel X **Other** (*specify*): **Non-Randomized, Single arm** |

| **Study Rationale**  Although the advent of combination antiretroviral therapy (ART) has dramatically improved the expectance and life quality of HIV-1 infected individuals, it has failed in terms of eradication of infection. Moreover, the need for HIV-infected subjects of a continuous ART intake that cannot be interrupted, is frequently associated with “fatigue”, a common problem which contributes to both morbidity and disability and explains why it frequently happens that patients ask for an antiretroviral “pause”.  Up to now, the studies investigating ART interruption have been mainly conducted on subjects with primary HIV infection and only few on chronically infected individuals without a known seroconversion date, although they represent the majority of the HIV-infected population.  Unfortunately, none of these studies has resulted in a successful outcome for most HIV infected patients, experiencing rebounding of virus replication and disease progression requiring the reintroduction of ART but they additionally highlighted that drug-free control of viral replication occurs in a minority of individuals.  The studies evaluating ART interruption are important because they may allow the understanding of the determinants of time to HIV rebound and the extent of CD4+ loss and then they may help in the search of a cure that may eliminate or control the HIV-reservoir.  The most recent studies have been designed with an intensive monitoring during the ART-free follow-up in the attempt to limit risks associated with ART interruption (viral rebound, CD4+ decrease, clinical events, resistance emergence, increased risk of HIV transmission) and ART is restarted as soon as viral load threshold is reached.  We hypothesize that patients with the highest chance of controlling HIV infection after ART withdrawal are those that, likely due to a favourable genetic background and a long-lasting viral suppression, have a smaller replication competent HIV reservoir, i.e. no measurable residual viremia and extremely low HIV-DNA load.  Therefore, we here propose a proof-of-concept study on 30 adult (>18 and <65 years old) chronically HIV-1 infected, ART-treated, with undetectable viremia for ≥10 years, undetectable HIV-DNA, CD4+≥500 cells/µL and no evidence of detectable residual viremia for ≥5 years to be enrolled in a protocol of Intensively Monitored Antiretroviral Pause (IMAP) up to 12 months of follow-up with a tight clinical and laboratory monitoring of their condition. The primary goal of the study is to explore the frequency of spontaneous control of viral replication after IMAP. The secondary goal will be the identification of virological, and immunological biomarkers associated with spontaneous control of viral replication after IMAP. |
| --- |
| **Study design**  Prospective, open-label, single arm, non-randomized, proof-of-concept study.  Eligible patients will sign a written informed consent and will be followed-up at screening, baseline (ART interruption) and at 1, 2, 3, 4, 6, 8, 10, 12, 16, 24, 32, 40, 48 weeks thereafter or at ART resumption.  The study visits will include: general clinical assessment, routine laboratory tests including: creatinine, phosphorus, calcium, alkaline phosphatase, AST, ALT, fasting glucose, total cholesterol, HDL- and LDL-cholesterol, triglycerides, CD4+ cell count and CD4+/CD8+ ratio.  Additional 30 mL of peripheral blood will be withdrawn at study visits for further virological, and immunological investigations and for bio-banking purposes.  During follow-up, the occurrence of two consecutive HIV-1 RNA values >50 copies/mL or the occurrence of stage B or C AIDS-defining events or any serious non-AIDS clinical event at least potentially related to treatment interruption will be criteria for ART resumption.  All patients with HIV-RNA<50 copies/mL at week 48 (end of the study) will resume their baseline ART regimen.  The main demographic, clinical and therapy information will be accurately recorded at the study visits in an electronic Case Report Form (eCRF). |
| **OBJECTIVES**  **Primary**  To explore the frequency of spontaneous control of viral replication after Intensively Monitored Antiretroviral Pause (IMAP) among adult (>18 and <65 years old) chronically HIV-1 infected subjects with undetectable viremia for ≥10 years, undetectable HIV-DNA, CD4+≥500 cells/µL and no evidence of detectable residual viremia for ≥5 years.  **Secondary**  To identify the virological and immunological markers associated with spontaneous control of viral replication after IMAP. |
| **ENDPOINTS**  **Primary**  Cumulative proportion of patients who will not resume ART 12 months after IMAP due to the occurrence of two consecutive HIV-1 RNA values >50 copies/mL or the occurrence of stage B or C AIDS-defining events or any serious non-AIDS clinical event at least potentially related to treatment interruption.  **Secondary**   - change from baseline in plasma viremia and HIV-DNA after IMAP at study visits or at ART resumption; - change in CD4+ counts, CD4+/CD8+ ratio since IMAP at study visits or at ART resumption. |
| **Main Inclusion/Exclusion Criteria**  **Inclusion criteria:**  The study will include HIV-1 infected:   - men and non-pregnant women, - ≥18 and <65 years-old, - asking to stop therapy, - with HIV-1 RNA<50 copies/mL for ≥10 years, - current CD4+≥500 cells/µL, - HIV-DNA<100 copies/10^6^PBMCs, - no evidence of detectable residual viremia for ≥5 years .   **Exclusion criteria:**  The study will exclude HIV-1 infected subjects:   - significant risk of HIV transmission during IMAP (including evidence of not adopting effective contraception methods and women who wish to be pregnant) in the opinion of the investigator, - pregnancy and breastfeeding, - a documented pre-ART HIV-1 RNA<200 copies/mL, - reactive Hepatitis B virus (HBV) surface antigen, - positive HCV-RNA at the time of screening, - current AIDS defining event as defined in category C of the 'Centers for disease control and prevention (CDC)' clinical classification, - previous diagnosis of diabetes, - a previous diagnosis of cancer or major adverse cardiac events (MACE) and currently receiving chemotherapy or immuno-modulating agents at the time of screening, - history of HIV-related thrombocytopenia, - active renal disease defined as a glomerular filtration rate (calculated by MDRD equation) below 50 mL/min or the presence of HIV associated nephropathy in the past medical history, - any condition, including psychiatric or psychological disorders that might interfere with adherence to study requirements or safety of the participant, - prior use of any HIV vaccine and/or non-established experimental therapy, - active drug or alcohol use or dependence that, in the opinion of the site investigator, would interfere with adherence to study requirements. |
| **Sample size calculation**  The ULTRASTOP Study showed that the probability of remaining with viremia <400 copies/mL, with CD4+>400 cells/µL and with no occurrence of CDC grade B or C clinical event 12 months after treatment interruption was 10% (95% CI: 0.3% to 44.5%) among chronic treated subjects with plasma HIV-RNA<50 copies/mL for more than 2 years on ART, CD4+>500 cells/µL, CD4+/CD8+ ratio>0.9, CD4+ nadir>300 cells/µL and HIV-DNA<100 copies/10^6^ peripheral blood mononuclear cells.  We estimated that a sample size of 134 subjects produces a 95% confidence interval with a precision of 5% under the null hypothesis that the proportion of subjects who will not resume ART 12 months after IMAP (primary endpoint) is 10% or less.  Among the 4771 patients currently followed at the Infectious Diseases Department (freezing date: 31th January 2016), 563 subjects maintained undetectable viremia (HIV-1 RNA< 50 copies/mL) for ≥10 years, 155 patients have no evidence of detectable residual viremia since at least 5 years and 131 subjects have current CD4+>500 cells/µL. Based on internal preliminary data, as we expect that 30% of these patients have HIV-DNA<100 copies/10^6^PBMCs, then a proof-of-concept study with a sample size of 30 patients is a feasible study. |
| **Statistical analysis**  The statistical analysis on either the primary endpoint and the secondary endpoints will consider all the subjects enrolled in the study consisting of those subjects who will have “received” at least one day of ART interruption.  An initial description of the enrolled subjects’ characteristics will be provided by use of the median and quartiles (25^th^ and 75^th^).  The cumulative proportion of patients who will not resume ART (primary endpoint) will be determined using Kaplan-Meier estimates.  Factors associated with the occurrence of the primary endpoint will be analysed using univariate and multivariate Cox proportional hazard models.  Changes from baseline in CD4+ cell count, CD4+/CD8+ ratio and the other laboratory parameters will be summarized at each time-point at which were collected with a major interest at ART resumption/12-months and assessed for significance by use of the Wilcoxon signed rank test.  As there are no pre-specified criteria for terminating the study early, no formal interim efficacy analysis is planned. However, results on the primary endpoint will be described and reported to the DSMB at 3, 6 and 12 months. The primary intent of these reports will be to monitor that the ART re-introduction will promptly lead to undetectable viral load or will effectively recover clinical events. In addition, the findings of the 3- and 6-month exploratory reports will be of help in evaluating the opportunity to further extend the study over 96 weeks. |

| **FLOW-CHART** | (screening & baseline) | | Follow-up (weeks) | | | | | | | | | | | | |
| --- | --- | --- | --- | --- | --- | --- | --- | --- | --- | --- | --- | --- | --- | --- | --- |
|  | -42 days | BL | 1 | 2 | 3 | 4 | 6 | 8 | 10 | 12 | 16 | 24 | 32 | 40 | 48 or ART resumption |
| Informed Consent | X |  |  |  |  |  |  |  |  |  |  |  |  |  |  |
| Fulfillment of the inclusion/exclusion criterias | X |  |  |  |  |  |  |  |  |  |  |  |  |  |  |
| Clinical assessment^§^ | X | X | X | X | X | X | X | X | X | X | X | X | X | X | X |
| Concomitant medications | X | X | X | X | X | X | X | X | X | X | X | X | X | X | X |
| HIV-1 RNA | X | X | X | X | X | X | X | X | X | X | X | X | X | X | X |
| Total HIV-DNA | X |  |  |  |  |  |  |  |  |  |  |  |  |  | X |
| Routine Lab.Tests^§§^ | X | X |  |  |  | X |  |  |  | X |  | X |  |  | X |
| Genotype^§§§^ |  |  |  |  |  |  |  |  |  |  |  |  |  |  | X |
| Plasma and PBMCs Storage | X | X | X | X | X | X | X | X | X | X | X | X | X | X | X |
| AE | X | X | X | X | X | X | X | X | X | X | X | X | X | X | X |
| SAE | X | X | X | X | X | X | X | X | X | X | X | X | X | X | X |

§ Clinical assessment includes the evaluation of CDC stage, height, weight, systolic and diastolic blood pressure and smoking status.

§§ Routine laboratory tests (hematological tests and biochemistry,) including creatinine, phosphorus, calcium, alkaline phosphatase, AST, ALT, fasting glucose, total cholesterol, HDL- and LDL-cholesterol, triglycerides, CD4+ cell count and CD4+/CD8+ ratio, urine analysis.

§§§ Genotype will be performed only in case of confirmed virological failure (HIV-1 RNA>50 copies/mL in two consecutive measurements).

**TABLE OF CONTENTS**

PROTOCOL TITLE PAGE 1

SYNOPSIS 2

FLOW-CHART OF THE STUDY 8

TABLE OF CONTENTS 9

1 INTRODUCTION 11

1.1 Introduction and Rationale 11

1.2 Aim of the Study 14

1.3 Risks and Benefits 14

2 STUDY OBJECTIVES AND ENDPOINTS 15

2.1 Primary Objective 15

2.2 Primary endpoint 15

2.3 Secondary Objectives 15

2.4 Secondary endpoints 15

3 STUDY DESIGN AND EVALUATION 16

3.1 Study Design 16

3.2 Definitions 16

3.3 Length of the study 17

3.4 Study Population 17

3.5 Inclusion Criteria 17

3.6 Exclusion Criteria 17

3.7 Methods of contraception 18

3.8 Concomitant medications/diet/activity 19

3.9 Discontinuation from the study 19

3.10 Sample collection and bio-banking 19

3.11 HIV-1 RNA and HIV-DNA assessment 19

4 METHODOLOGY 20

4.1 Sample Size 20

4.2 Statistical Analysis Plan 21

5 STUDY PROCEDURES 22

5.1 Ethical Approval 22

5.2 Responsibilities within the Study 22

5.3 Reports and Publications 25

5.4 Adverse Event Reporting 25

5.5 Data Collection, Database Retention and Archiving 30

5.6 Indemnity and compensation in case of damage 30

5.7 Data Safety Monitoring Board (DSMB) 30

6 LIST OF ABBREVIATIONS 31

7 REFERENCES 32

8 APPENDIXES (1-2-3-4-5-6) 34-90

**INTRODUCTION**

**1.1 Introduction and Rationale**

Combination antiretroviral therapy (ART), when optimally pursued, turns-off HIV-1 replication down to undetectable levels in plasma (<50 copies of HIV-1 RNA/mL) and restores the immune system, at least in part, making HIV-1+ individuals resistant to opportunistic infections (OI) and cancers, the hallmarks of full-blown AIDS occurring in 95% of infected individuals not treated with ART [1]. However, the need for HIV-infected subjects of a continuous ART intake that cannot be interrupted, is frequently associated with the “patient’s fatigue”, a common problem which contributes to either morbidity or disability and explains why it frequently happens that patients ask for an antiretroviral “pause”.

Up to now, the studies investigating ART interruption have been mainly conducted on subjects with primary HIV infection and only few on chronically infected individuals without a known seroconversion date, although they represent the majority of the HIV-infected population. These studies failed to lead to satisfactory results both in terms of therapeutic management or of pharmaco-economical considerations as the savings from a lower use of drugs was offset by the cost of testing and monitoring during recovery phase of treatment [2-5]. However, they highlighted that drug-free control of viral replication occurs in a minority of individuals.

A French study [6] reported that ca. 15% of patients maintained a spontaneous control of viremia and good healthy conditions for a median of 75 months among 72 individuals who initiated ART within 6 months since primary infection, remained on successful therapy for a median of 5 years and then interrupted the assumption of ART for different reasons.

The SPARTAC trial on PHI patients [7], found that total HIV-1 DNA was a predictor of plasma viral load rebound following therapy interruption. In addition, unspliced and spliced HIV-RNA species are produced contributing to the residual viremia detected in plasma [8]. Therefore, the measurement of cell-associated HIV-DNA (CAD) and HIV-RNA (CAR) is of pivotal importance in understanding HIV persistence.

Immunological biomarkers can predict time to viral rebound after stopping ART. A very recent study [9] demonstrated that T cell exhaustion markers including PD1, Tim-3 and Lag-1, evaluated during primary infection and after ART interruption, identify latently infected cells with a higher trend to viral transcription.

Latent viral infection has been also associated with a prolonged HIV antibody response [10]. The serum antibody levels may reflect the total body reservoir and this response is also stable during long-term ART [11]. Very recent studies showed an association between anti-gp41 Env and latent viral infection13 and between total and integrated HIV-DNA and antibody levels against Env and Pol proteins [12].

With regard to chronically HIV-infected subjects with undefined time of seroconversion, very recent studies have started to report findings on time to HIV rebound after treatment interruption and its association with some key measures of HIV reservoir in chronically HIV-infected subjects [13-15]. A French study [13] showed that 7.5% (7/95) of chronic HIV-infected subjects with pre-ART CD4+>350 cells/uL and plasma HIV-RNA<50,000 copies/mL and treated for a median of 5.3 years maintained a spontaneous control of their viremia levels <400 copies/mL for 12 months or longer after ART interruption. In this study, the multivariate analysis showed that HIV-DNA was the only predictive factor of loss of viral control while CD4 nadir didn’t.

The ULTRASTOP Study [14], another French proof-of-concept study with more severe selection criteria, showed that 10% (1/10) of chronic HIV-infected subjects with plasma HIV-RNA<50 copies/mL for more than 2 years on cART, CD4+>500 cells/µL, CD4+/CD8+ ratio>0.9, CD4+ nadir>300 cells/µL and HIV-DNA<100 copies/10^6^ peripheral blood mononuclear cells (PBMCs) undergoing treatment interruption maintained viral load <400 copies/mL and CD4+>400 cells/µL and no CDC grade B or C clinical event after 24 weeks following ART interruption. Importantly, this study also showed that suppressive therapy can be safely interrupted with rapid return to pre-interruption values and no relevant clinical or virological damage and no increase in cell-blood reservoir after treatment resumption.

A recent cross-sectional study [15] on 522 chronically infected patients on long-term suppressive ART (median 5.7 years) indicated that each additional year with suppressed HIV-RNA increased the likelihood of having HIV DNA low levels (<150 copies/10^6^ PBMCs) while it decreased the likelihood of high HIV-DNA levels (>1,000 copies/10^6^ PBMCs).

In addition, Zheng L et al. [16] found that longer duration of viral suppression were associated with lower levels of residual viremia (median 0.2 vs 2.3 copies/mL) and that residual viremia, in turn, is predictive of viral rebound [17-18]. The evidence that the presence of a detectable plasma residual viremia is associated with a shorter time to HIV rebound of at least 200 HIV-1 RNA copies/mL (≤4 vs 5-8 vs ≥8 weeks: 47% vs 29% vs 8%, p=0.02) was also reported in another study from ACTG [19], including 155 chronic patients.

The ISALA Study (NCT02590354) is an ongoing study recruiting 45 chronic HIV-1 infected patients, on ART since at least 2 years before baseline with no changes in the ART regimen for at least 90 days prior to study entry, with nadir CD4+ T-cell count is ≥300/μl, CD4+ T-cell count >= 500/μl for a period of at least 3 months prior study entry and undetectable viral load (plasma HIV-1 RNA < 50 copies/mL for at least 2 years before baseline (occasional "blips" are permitted if it happened more than six months before study entry) in four Belgian HIV reference centers. In this study, no randomization is foreseen. Patients will receive an intense clinical and laboratory follow-up during 48 weeks followed by 12 weeks post intervention.

Studies on treatment interruption raise safety and informed consent issues because treatment interruptions are not generally recommended in treatment guidelines; this is largely because the Strategies for the Management of Antiretroviral Therapy (SMART) study found that stopping treatment increases the risk of serious and fatal illness compared to continuous therapy [20]. However, these studies may allow the understanding of the determinants of time to HIV rebound and the extent of CD4+ loss and then are important in the search of a cure that may eliminate or control the HIV-reservoir; they also need to be designed in consideration of previous findings in the attempt to reduce patients’ risks and avoid unlikely results of viral control.

Although limited in number and with many differences in their eligibility criteria, previous studies evaluating the effect of ART treatment interruption in chronic HIV-infected patients, showed that potential predictive factors of HIV rebound are: length of viral suppression, nadir and current CD4+, total HIV-DNA.

Then, the study design we here propose:

- defined much more restrictive inclusion selection criteria than those considered in previous trials on chronic HIV-infected patients [13-14] or on subjects with primary HIV infection [20], aimed at minimizing risks of adverse outcomes to participants;
- exclude the enrollment of HIV-infected subjects with hepatitis B or C coinfection [21];
- exclude the enrollment of patients with comorbidities such as diabetes/cardiovascular disease/cancer which might be impaired by the occurrence of detectable viral load [22];
- planned an intensive monitoring of enrolled patients so that the restart of ART will occur as soon as viral load becomes detectable during the study with the aim of limiting the magnitude of viral load rebound and decline in CD4+ T cell count;
- include patients who ask to interrupt ART;
- planned to enroll a small sample limiting the number of patients who will be exposed to ART interruption and to its potential consequences.

**1.2 Aim of the Study**

The aim of this proof-of-concept study on adult (>18 and <65 years old), chronically HIV-1 infected individuals with undetectable viremia for ≥10 years, undetectable HIV-DNA, CD4+≥500 cells/µL and no evidence of detectable residual viremia for ≥5 years is to evaluate the frequency of spontaneous control of virus replication after ART pausing for up to 12 months and to identify the virological and immunological markers associated with spontaneous control of viral replication.

**1.3 Risks and Benefits**

The use of ART is associated with several limitations such as toxicity, adverse effects, lack of adherence and drug-drug interactions so that patients often “suffer” from the need to be continuously treated and ask for a “pause”. Thus, a study evaluating ART interruption among HIV-1 infected patients, successfully treated for a very long time during the chronic phase of HIV infection will tell us that the spontaneous control of viral replication is possible even during the chronic phase of HIV infection and not only in the case of very early treatment in primary HIV infection. This study might also help in suggesting markers predictive of ART-free spontaneous viral control which may be useful for future larger IMAP studies.

The risks associated with ART interruption include: viral rebound, acute retroviral syndrome, increased risk of HIV transmission, decline of CD4+ count, HIV disease progression, development of a serious non-AIDS complications (e.g. renal, cardiac, hepatic, or neurologic complications) and of drug resistance.

Details regarding potential risks for subjects enrolled in this study may be found in the Informed Consent document.

**2 STUDY OBJECTIVES AND ENDPOINTS**

**2.1 Primary Objective**

To explore the frequency of spontaneous control of viral replication after Intensively Monitored Antiretroviral Pause (IMAP) [19] among adult (>18 and <65 years old), chronically HIV-1 infected subjects with undetectable viremia for ≥10 years, undetectable HIV-DNA, CD4+≥500 cells/µL and no evidence of detectable residual viremia for ≥5 years.

**2.2** **Primary endpoint**

Cumulative proportion of patients who will not resume ART 12 months after IMAP due to the occurrence of two consecutive HIV-1 RNA values >50 copies/mL or the occurrence of stage B or C AIDS-defining events or any serious non-AIDS clinical event at least potentially related to treatment interruption.

**2.3 Secondary Objectives**

To identify virological and immunological markers associated with spontaneous control of viral replication after IMAP.

**2.4 Secondary endpoints**

- change from baseline in plasma viremia and HIV-DNA after IMAP at study visits or at ART resumption;
- change in CD4+ counts, CD4+/CD8+ ratio since IMAP at study visits or at ART resumption.

**3 STUDY DESIGN**

**3.1 Study Design**

Prospective, open-label, single arm, non-randomized, proof-of-concept study.

Eligible patients will sign a written informed consent and will be followed-up at screening, baseline (ART interruption) and at 1, 2, 3, 4, 6, 8, 10, 12, 16, 24, 32, 40, 48 weeks thereafter or at ART resumption.

The clinical assessment (study visit) will include: the evaluation of CDC stage, height, weight, systolic and diastolic blood pressure and smoking status, routine laboratory tests [including creatinine, phosphorus, calcium, alkaline phosphatase, AST, ALT, fasting glucose, total cholesterol, HDL- and LDL-cholesterol, triglycerides, CD4+ cell count and CD4+/CD8+ ratio, urine analysis].

Additional 30 mL of peripheral blood will be withdrawn at study visits and stored in a biobank for further investigations.

During follow-up, the occurrence of two consecutive HIV-1 RNA values >50 copies/mL or the occurrence of stage B or C AIDS-defining events will be criteria for ART resumption or any serious non-AIDS clinical event at least potentially related to treatment interruption.

All patients with HIV-RNA<50 copies/mL at week 48 (end of the study) will resume their baseline ART regimen.

The main demographic, clinical and therapy information will be accurately recorded at the study visits in an electronic Case Report Form (eCRF).

**3.2 Definitions**

Confirmed virological rebound (CVR) is defined as the occurrence of two consecutive HIV RNA ≥50 copies/mL while on study.

Residual viremia will be defined as any detectable HIV-RNA value below 50 copies/mL.

**3.3 Length of Study**

Subjects will complete the study when they will resume ART or reach week 48.

The last patient visit is the last on-study visit or date of death or lost-to follow-up.

**3.4 Study Population**

This study will include adult (>18 and <65 years old) HIV-1 infected patients, on treatment with any type of antiretroviral regimen, asking to stop therapy and who meet the other inclusion criteria described in this protocol.

All patients will sign an informed consent to participate the study.

A total of 30 patients is planned to be enrolled.

**3.5 Inclusion Criteria**

The study will include HIV-1 infected:

- men and non-pregnant women,
- ≥18 and <65 years-old,
- asking to stop therapy,
- with HIV-1 RNA<50 copies/mL for ≥10 years,
- current CD4+≥500 cells/µL,
- HIV-DNA<100 copies/10^6^PBMCs,
- no evidence of detectable residual viremia for ≥5 years .

**3.6 Exclusion Criteria**

The study will exclude HIV-1 infected subjects with:

- significant risk of HIV transmission during IMAP (including evidence of not adopting effective contraception methods and women who wish to be pregnant) in the opinion of the investigator,
- pregnancy and breastfeeding,
- a documented pre-ART HIV-1 RNA<200 copies/mL,
- reactive Hepatitis B virus (HBV) surface antigen,
- positive HCV-RNA at the time of screening,
- current AIDS defining event as defined in category C of the 'Centers for disease control and prevention (CDC)' clinical classification,
- previous diagnosis of diabetes,
- a previous diagnosis of cancer or major adverse cardiac events (MACE) and currently receiving chemotherapy or immuno-modulating agents at the time of screening,
- history of HIV-related thrombocytopenia,
- active renal disease defined as a glomerular filtration rate (calculated by MDRD equation) below 50 mL/min or the presence of HIV associated nephropathy in the past medical history,
- any condition, including psychiatric or psychological disorders that might interfere with adherence to study requirements or safety of the participant,
- prior use of any HIV vaccine and/or non-established experimental therapy,
- active drug or alcohol use or dependence that, in the opinion of the site investigator, would interfere with adherence to study requirements.

**3.7 Methods of contraception**

Site personel (physician or nurse) will verify that enrolled subjects (men and women) and their partners use methods of contraception while on ART interruption.

Enrolled men and women (or their partner) will need to agree to use contraception methods and to not participate in a conception process (e.g., active attempt to become pregnant, sperm donation, or in vitro fertilization) during the study.

Enrolled females of reproductive potential will need a negative serum or urine pregnancy test at screening and agree to avoid becoming pregnant during the study and for at least 15 days after study completion; females of reproductive potential are defined as those who have not reached menopause or been post-menopausal for at least 24 consecutive months, who have had menses within the preceding 24 months, or women who have not undergone surgical sterilization (hysterectomy and bilateral oophorectomy, bilateral salpingectomy, tubal micro-inserts).

Vasectomy of male subjects will also be considered as a valid contraception method.

Patient’s consent to use acceptable methods of contraception will be documented in the subject’s chart.

**3.8** **Concomitant medications/diet/activity**

The use of concomitant medications/therapies/diet/activity (already ongoing or that might need to be prescribed during the study) are allowed.

No specific restrictions are foreseen during the course of the study with regard to concomitant therapy or vaccination or diet requirements or physical activity.

**3.9** **Discontinuation from the study**

Patients may withdraw consent at any time for any reason or be dropped from the study at discretion of the investigator if he/she violates the study plan or for administrative or other safety reasons.

Subjects will be discontinued from the study if they will:

- withdraw consent or
- resume ART for the occurrence of a confirmed virological rebound or of a stage B or C AIDS-defining event or any serious non-AIDS clinical event at least potentially related to treatment interruption.

Any female patient who becomes pregnant during the course of the study will be also immediately withdrawn from the study.

**3.10 Sample collection and bio-banking**

Enrolled patients will be asked to sign a specific informed consent for the collection of additional plasma and PBMCs samples at the scheduled study visits in order to perform further virological and immunological investigations.

In these patients, 30 mL of peripheral blood will be additionally collected at each study visit.

**3.11 HIV-1 RNA and HIV-DNA assessment**

HIV-1 RNA will be quantified by the kinetic PCR molecular system (Abbott Real Time PCR) which gives three possible outputs: HIV-RNA>40 copies/mL (quantitative result); HIV-RNA≤40 copies/mL (semi-quantitative result: HIV-RNA is detectable but not quantifiable); undetectable viraemia (qualitative result: no signal can be detected).

HIV-DNA will be quantified by Real Time PCR (ABI Prism 7900).

**4. METHODOLOGY**

**4.1 Sample size**

The ULTRASTOP Study [14] showed that the probability of remaining with viremia <400 copies/mL, with CD4+>400 cells/µL and with no occurrence of CDC grade B or C clinical event 12 months after treatment interruption was 10% (95% CI: 0.3% to 44.5%) among chronic treated subjects with plasma HIV-RNA<50 copies/mL for more than 2 years on ART, CD4+>500 cells/µL, CD4+/CD8+ ratio>0.9, CD4+ nadir>300 cells/µL and HIV-DNA<100 copies/10^6^ peripheral blood mononuclear cells.

We estimated that a sample size of 134 subjects produces a 95% confidence interval with a precision of 5% under the null hypothesis that the proportion of subjects who will not resume ART 12 months after IMAP (primary endpoint) is 10% or less, as shown in the table below.

| Estimated 12-month proportion of pts with no ART resumption after IMAP | Precision | | | | |
| --- | --- | --- | --- | --- | --- |
|  | 5% | 6% | 7% | 8% | 9% |
| 6% | 73 | - | - | - | - |
| 7% | 84 | 62 | - | - | - |
| 8% | 108 | 72 | 54 | - | - |
| 9% | 115 | 80 | 50 | 48 | - |
| 10% | 134 | 88 | 59 | 45 | 37 |

Among the 4771 patients currently followed at the Infectious Diseases Department (freezing date: 31th January 2016), 563 subjects maintained undetectable viremia (HIV-1 RNA< 50 copies/mL) for ≥10 years, 155 patients have no evidence of detectable residual viremia since at least 5 years and 131 subjects have current CD4+>500 cells/µL. Based on internal preliminary data, as we expect that 30% of these patients have HIV-DNA<100 copies/10^6^PBMCs, then a proof-of-concept study with a sample size of 30 patients is a feasible study.

**4.2 Statistical Analysis Plan**

The statistical analysis on either the primary endpoint and the secondary endpoints will consider all the subjects enrolled in the study consisting of those subjects who will have “received” at least one day of ART interruption.

An initial description of the enrolled subjects’ characteristics will be provided by use of the median and quartiles (25^th^ and 75^th^).

The cumulative proportion of patients who will not resume ART (primary endpoint) will be determined using Kaplan-Meier estimates.

Factors associated with the occurrence of the primary endpoint will be analysed using univariate and multivariate Cox proportional hazard models.

Changes from baseline in CD4+ cell count, CD4+/CD8+ ratio and the other laboratory parameters will be summarized at each time-point at which were collected with a major interest at ART resumption/12-months and assessed for significance by use of the Wilcoxon signed rank test.

Missing values due to a skipped visit or to an inadequate sample will be:

- allowed if intermittent (i.e followed at the subsequent study visit by observed values), and not considered to be suggestive of CVR or occurrence of stage B or C AIDS-defining events or any serious non-AIDS clinical event at least potentially related to treatment interruption;
- considered as failure [Non-Completer=Failure (NC=F) approach] if missing values will be observed in at least two consecutive visits.

Nominal p-values will be computed in the analyses but should be interpreted with caution due to the issues of study design (proof-of-concept study) and sample size. Statistical tests will be performed considering a two-sided alpha level of 0.05 as reference to detect statistical significance.

As there are no pre-specified criteria for terminating the study early, no formal interim efficacy analysis is planned. However, results on the primary endpoint will be described and reported to the DSMB at 3, 6 and 12 months. The primary intent of these reports will be to monitor that the ART re-introduction will promptly lead to undetectable viral load or will effectively recover clinical events. In addition, the findings of these exploratory reports will be of help in evaluating the opportunity to further extend the study over 96 weeks.

All analyses will be performed with SAS Software, release 9.2.

**5 STUDY PROCEDURES**

**5.1 Ethical approval**

In accordance with the regulations in force, the Investigator must obtain approval from the appropriate Regulatory Authority before starting the clinical study.

This study will be conducted in accordance with the ethical principles that have their origin in the current Declaration of Helsinki (see appendix 2) and will be consistent with International Conference on Harmonization Good Clinical Practice (ICH GCP), Good Epidemiology Practices (GEP), and applicable regulatory requirements.

Prior to initiation of the trial at any site, the trial, including the protocol, informed consent, and other trial documents must be approved by an appropriate Institutional Review Board (IRB) or Independent Ethics Committee (IEC). The IRB/IEC must be constituted according to applicable regulatory requirements. The investigator must guarantee that the protocol has been seen and approved by the or before starting the study.

As appropriate, amendments to the protocol must also be approved by the IRB/IEC before implementation at the site. The IRB/IEC approval should be obtained in writing, clearly identifying the trial, the document reviewed (including informed consent), and the date of the review.

Until such time, it will be necessary to refer to the previous version of the already approved document.

**5.2 Responsibilities within the study**

The study shall be conducted as described in this approved protocol. All revisions to the protocol must be discussed with, and be prepared by, the principal investigator. The authors should not implement any deviation or change to the protocol without prior review.

**5.2.2 Informed Consent Form**

The investigator or other designated personnel have the task of informing the subjects about all of the aspects and procedures of the study.

The process of obtaining informed consent must comply with the regulatory procedures in force. The investigator (or a designated collaborator) and the subject must date and sign the informed consent form before the patient starts any procedure relating to the study. The subject will receive a copy of the ICF dated and signed by both parties; the original copy will be kept in the study archives. Neither the investigator nor the designated personnel should in any way exercise any coercion or influence on a subject to induce him/her to participate or continue to participate in the study. The decision of a subject to participate in the study must be completely voluntary. The investigator and designated personnel must emphasise to the subject that he/she can withdraw his/her consent at any time without any penalty or loss of any benefits to which he/she may be entitled.

The written or oral information concerning the study, including the written consent form, must not contain any language that forces the subject to renounce (even only apparently) his/her legal rights, or which would exonerate the investigator, institution or sponsor from liability for negligence.

**5.2.3 Duties of the Investigator**

In accordance with the applicable local regulations, the investigator shall make periodic reports concerning the progress of the study in his/her centre to the IEC/IRB, and notify the same at study closure. The periodic reports and closure notification are part of the responsibilities of the Investigator.

**5.2.4 Study Monitoring**

In accordance with the applicable regulations and Good Clinical Practice (GCP), the monitor shall periodically visit or contact the centre. The duration, nature and frequency of such visits/contacts shall depend on the rate of recruitment, the quality of the documents in the possession of the centre, and its adherence to the protocol.

Through these contacts, the monitor must:

- control and evaluate the progress of the study,
- examine the collected data,
- conduct Source Document Verification (SDV),
- identify every problem and find solutions.

The aims of the monitoring activity are to verify that:

- the rights and well-being of the subject are respected,
- the study data are accurate, complete and verifiable by original documents,
- the study is conducted in accordance with the protocol and any approved amendments, GCP and the applicable regulations.

The investigator must consent to:

- giving the monitor direct access to all relevant documentation;
- dedicating part of his/her time and the time of his/her staff to the monitor to discuss the results of the monitoring and any other possible aspects.

The monitor must also contact the centre before the beginning of the study to discuss the protocol and data collection procedures with the staff.

**5.2.5 Quality assurance**

In its capacity as promoter, San Raffaele Hospital can carry out a quality control audit at its discretion. In this case, the investigator must consent to giving the auditor direct access to all of the relevant documentation and dedicating part of his/her time and the time of his/her staff to the auditor, to discuss the results of the monitoring and any other possible aspects.

Furthermore, the Regulatory Authorities may also inspect the study. In this case, the investigator must consent to giving the inspector direct access to all of the relevant documentation and dedicating part of his/her time and the time of his/her staff to the inspector to discuss the results of the monitoring and any other possible aspects.

**5.2.6 Trial and Centre closure**

At the time of study closure, the monitor and the investigator must activate a series of procedures:

- review all the study documentation
- reconcile the study data
- reconcile any clarification reports

**5.2.7 Trial Documents and Records Retention**

During the trial and after termination of the trial, including after early termination, the investigator must maintain copies of all documents and records relating to the conduct of the trial. This documentation includes, but is not limited, to protocols, Case Report Forms (CRFs), adverse event reports, subject source data, correspondence with health authorities and IRB/IECs, informed consent form, investigator’s curricula vitae, monitor visit logs, laboratory reference ranges and laboratory certification or quality control procedures . The investigator must retain trial records for the amount of time specified by applicable local laws and regulations. At a minimum , trial records must be retained for the amount specified by ICH Guidelines or EU GCP, whichever is longer:

- The ICH Guidelines specify that records must be retained for a minimum of 2 years after a marketing application for the indication is approved or 2 years after notifying the appropriate regulatory agency that an investigation is discontinued
- The EU GCP Directive specifies that trial records must be retained for 5 years after the completion of the trial.

**5.3 Reports and Publications**

The confidentiality of records that could identify subjects within the database must be protected, respecting the privacy and confidentiality rules in accordance with the applicable regulatory requirement(s). Documentation and archiving of the database may be implemented. Publication plan should be identified and prepared by the principal investigator with the involvement of participating centres.

**5.4 Adverse Events Reporting**

All adverse events identified during this study must be reported to the competent authority in accordance with current local regulations and laws. Notification will take place within 24 hours of the investigator becoming aware of the event.

A description of any adverse events will be notified to the Designee for the Pharmacovigilance of the study (Prof. Antonella Castagna tel +39 02 26437903).

**5.4.2 Assessment and reporting Adverse events (AEs) and Serious Adverse Events (SAes)**

The investigator is responsible for reporting and documenting events falling within the protocol definitions of AEs or SAEs. During the treatment period, the investigator or designated sub-investigator shall be responsible for reporting AEs and SAEs as described in this section of the protocol. In order to satisfy international safety requirements, the investigator must include in his/her evaluation every SAE caused by participation in the study (e.g. any complications arising from blood sampling).

**5.4.3 Definition of an Adverse Event (AE)**

Per GCP, an adverse event is any untoward medical occurrence in a patient irrespective of study treatment status. An adverse event (AE) can therefore be any unfavourable and unintended sign (including an abnormal laboratory finding), symptom, or disease whether or not related to the study treatment status.

**5.4.4 Assessment of Adverse Event Severity and Relationship to Treatment Status**

The Division of AIDS (DAIDS) in the United States has developed a graduated scale to evaluate the severity of Adverse Events and laboratory abnormalities of clinical significance during the study participation. The investigator must utilize these definitions whenever possible. For abnormalities not reported on the Toxicity scale please refer to the scale reported on appendix 1 which defines the grade of severity of the event.

**Grade 1 (Mild):** Symptoms causing no or minimal interference with usual social and functional activities.

**Grade 2 (Moderate):** Symptoms causing greater than minimal interference with usual social and functional activities.

**Grade 3 (Severe):** Symptoms causing inability to perform usual social and functional activities.

**Grade 4 (Life threatening):** Symptoms causing inability to perform basic self-care functions OR Medical or operative intervention indicated to prevent permanent impairment, persistent disability, or death.

**5.4.5 Definition of a Serious Adverse Event (SAE)**

Per GCP a Serious Adverse Event is any untoward medical occurrence that:

- results in death,
- is life-threatening
- requires inpatient hospitalization or prolongation of existing hospitalization
- results in persistent or significant disability/incapacity, or is a congenital anomaly/birth defect.

Additionally, important medical events that may not results in death, be lifethreatening, or require hospitalization may be considered a serious adverse event when, based upon appropriate medical judgment, the may jeopardize the subject and may require medical or surgical intervention to prevent one of the outcomes listed in this definition. Example of such medical events include allergic bronchospasm requiring intensive treatment in an emergency room or at home, blood dyscrasias or convulsions that do not result in in-patient hospitalization, or the development of drug dependency or drug abuse.

SAEs that occur at any time after the inclusion of the subject in the trial up to 30 days after the subject completed or discontinued the trial must be reported. In the specific circumstance of screening failures, SAEs must be collected from the time of consent signing until the subject is considered a screen failure.

**5.4.6 Reporting of Subject Death**

The death of any subject after enrolment or within 30 days of trial completion, regardless of the cause, must be recorded by investigator or qualified designee within 1 working day of first becoming aware of the death. Report of death must be communicated as soon as possible to the appropriate local IRB.

**5.4.7 Pre planned Hospitalizations or Procedures**

During the trial if a subject has a hospitalization or procedure (eg, elective surgery) that was scheduled prior to the subject entering the trial (ie before the subject [or the subject’s legal representation] signed the informed consent) for an event/condition that occurred before the trial, the hospitalization is considered a therapeutic intervention and not the result of a SAE. However , if event/condition worsens during the trial, it must be reported as an AE (or SAE, if the event/condition results in a serious outcome such as hospitalization).

**5.4.8 Abnormal Laboratory Parameters and Other Abnormal Values as AEs or SAEs**

Abnormal laboratory values (e.g. biochemistry, haematology, urine test) or other abnormal clinical examinations (e.g. ECG, radiological examinations, vital signs) that are judged by the investigator to be clinically significant must be considered AEs or SAEs if they satisfy the respective definition criteria.

Clinically significant abnormal laboratory values or other signs diagnosed after the administration of the study intervention, or which were already present at the baseline visit and worsened after the start of the study, are considered AEs or SAEs. The investigator is obliged to exercise his/her medical and scientific judgement in deciding whether the abnormal laboratory values or other abnormal clinical examinations are or are not clinically significant.

**5.4.9 Reporting AEs and SAEs in the e-CRF**

All AEs and SAEs must be recorded in the study e-CRF and in the subject’s medical records.

All information to be reported for each SAE are contained in specific pages of CRFs.

If there is any change in the information over time, an updated SAE report must be sent.

**5.4.10 Documentation Relating to AEs and SAEs**

Each AE and SAE that occurs during the study must be documented in the medical records of the patient in accordance with the standard clinical practice of the investigator, and on the Adverse Events/SAE page of the e-CRF. A separate set of SAE pages must be used for each SAE. Only if, at the moment of the initial report, multiple SAEs are present and time and/or clinically inter-related is it possible to record them on the same SAE page.

The investigator should try to formulate a diagnosis of the event on the basis of signs, symptoms, and/or other clinical information. In these cases the diagnosis and not the individual signs/symptoms should be recorded on the AE and/or SAE pages.

If clinically significant abnormal laboratory values or other clinical examinations are consistent with the definition of an AE or SAE, the AE or SAE pages in the e-CRF must be appropriately completed. In brief, AE or SAE pages must be completed for each diagnosis (if known), clinical symptom/sign, or abnormal laboratory parameter of clinical significance. Laboratory data can be recorded in the SAE pages in two ways: 1) by directly inserting them in the SAE form with their normal values and baseline values of the subject; 2) by sending copies of the laboratory report accompanied by the SAE pages.

The SAE forms in the e-CRF must be completed as accurately as possible, printed and signed by the Investigator, before being sent to the Ethics Committee of San Raffaele Hospital. It is very important that the investigator provides his/her judgment regarding the relationship of the event to the study therapy on the initial SAE form.

**5.4.11 Follow-up of AEs or SAEs**

All AEs and SAEs must be followed up:

- until their complete resolution
- until their stabilisation
- until the event can be attributed a new aetiology
- until the patient ceases to be in the care of the Centre

The investigator must ensure that the follow-up reports include all supplementary information allowing a complete evaluation of the nature and/or the cause-effect relationship of the AE or SAE, including further laboratory and other tests, pathology reports, and any specialist examinations.

**5.4.12 Deadlines for SAE notification**

All SAEs must be reported by the investigator or qualified designee to Promoter (see box below) within 1 working day of first becoming aware of the event.

All SAEs should be also reported to local Ethic Committee.

Pharmacovigilance aspects will be conducted according to Appendix 3 described in this protocol.

NOTIFICATION OF SERIOUS ADVERSE EVENTS:

Prof. Antonella Castagna (Study Promoter & Designee for the Pharmacovigilance)

Department of Infectious Diseases, San Raffaele Hospital

FAX # : 02/2643.7903

**5.5 Data Collection, Database Retention and Archiving**

**5.5.1 Data Collection**

Electronic Data Capture (EDC) will be used for this trial, meaning that all Case Report Form data will be entered in electronic forms at the investigational site. The e-CRF has to be completed as soon as possible during or after the subject visit. The investigator must verify that all data entered are accurate and correct. The e-CRF will be compared with the source documents to ensure that there are no discrepancies between critical data. All entries, corrections and alterations have to be made by investigator or his/her designee.

**5.5.2 Database Retention and Archiving**

Location of database and supporting documentation will be outlined in the final report, in any case it is a responsibility of principal investigator.

**5.6 Indemnity and compensation in case of damage**

In accordance with the Italian Decreto Ministeriale dated 14/07/2009 an adequate insurance policy will cover for any issues regarding the indemnity and compensation in the case of damages for all the subjects enrolled in the study.

**5.7** **Data Safety Monitoring Board (DSMB)**

An independent data-monitoring board (DSMB) will be established to assess at intervals the progress of the study, the efficacy endpoints, the safety data and to recommend whether to continue, modify or stop the study.

**6 LIST OF ABBREVIATIONS**

| **Term** | **Definition** |
| --- | --- |
| IMAP | Monitored Antiretroviral Pause |
| AE | Adverse Event |
| AIDS | Acquired Immunodeficiency Syndrome |
| ART | Anti-retroviral therapy |
| CAD | cell-associated HIV-DNA |
| CAR | cell-associated HIV-RNA |
| CDC | Centre for Disease Control |
| GCP | Good Clinical Practice |
| GEP | Good Epidemiological Practice |
| HAART | Highly Active Antiretroviral Therapy |
| HBV | Hepatitis B virus |
| HCV | Hepatitis C virus |
| HDL | High Density Lipoproteins |
| IMAP | Intensively Monitored Antiretroviral Pause |
| LDL | Low Density Lipoproteins |
| HIV | Human Immunodeficiency Virus |
| IAS | International AIDS society |
| ICH | International conference on harmonization |
| OI | Opportunistic infection |
| RNA | Ribose nucleic acid |
| TI | Therapy interruption |

**7 REFERENCES**

1. Pau AK et al. Antiretroviral therapy: current drugs. Infect Dis Clin North Am 2014; 28: 371-402.
2. El-Sadr et al. CD4+ count-guided interruption of antiretroviral treatment. NEJM 2006; 355: 2283-2296.
3. Emery, S. et al. Major clinical outcomes in antiretroviral therapy (ART)-naive participants and in those not receiving ART at baseline in the SMART study. J Infect Dis 2008; 197: 1133-1144.
4. Skiest, Dj et al. Improved measures of quality of life, lipid profile, and lipoatrophy after treatment interruption in HIV-infected patients with immune preservation: results of ACTG 5170. J Acquir Immune Defic Syndr 2008; 49: 377-383.
5. Lundgren, JD et al. Inferior clinical outcome of the CD4+ cell count-guided antiretroviral treatment interruption strategy in the SMART study: role of CD4+ Cell counts and HIV RNA levels during follow-up. J Infect Dis 197: 1145-1155.
6. Saez-Cirion A et al. Post-treatment HIV-1 controllers with a long-term virological remission after the interruption of early initiated antiretroviral therapy ANRS VISCONTI Study. PLoS pathogens 2013; 9: e1003211.
7. Williams, JP et al. HIV-1 DNA predicts disease progression and post-treatment virological control. Elife 2014; 3: e03821.
8. Ho YC et al. Replication-competent noninduced proviruses in the latent reservoir increase barrier to HIV-1 cure. Cell. 2013; 155:540-51.
9. Hurst J et al. Immunological biomarkers predict HIV-1 viral rebound after treatment interruption. Nat Commun 2015; 6: 8495.
10. Lee S. Anti-HIV antibody responses reflect the quantifiable HIV reservoir size. IAS, Vancouver, Canada, 19-22 July 2015.
11. Siliciano JD et al. Long-term follow up studies confirm the stability of the latent reservoir for HIV-1 in resting CD4+ Tcells. Nat Med, 9:727-8, 2003.
12. Burbelo et al. Luciferase immunioprecipitation systems for measuring antibodies in autoimmune and infectious diseases. Transl Med, 165:325-35, 2015.
13. Assoumou L et al. A low HIV-DNA level in peripheral blood mononuclear cells at antiretroviral treatment interruption predicts a higher probability of maintaining viral control. AIDS 2015; 29: 2003-7.
14. Calin R et al. Treatment interruption in chronically HIV-infected patients with an ultralow HIV reservoir: results from the ULTRASTOP study. AIDS 2016, 29: 000-000.
15. Cuzin L et al. Levels of intracellular HIV-DNA in patients with suppressive antiretroviral therapy. AIDS 2015, 29: 1665-1671.
16. Zheng L et al. Predictors of residual viraemia in patients on long-term suppressive antiretroviral therapy. Antivir Ther 2013 18: 39-43.
17. Maggiolo F et al. Ultrasensitive assessment of residual low-level HIV viremia in HAART-treated patients and risk of virological failure. J Acquir Immune Defic Syndr 2012; 60:473-82.
18. Gianotti N et al. HIV DNA loads, plasma residual viraemia and risk of virological rebound in heavily treated, virologically suppressed HIV-infected patients. Clin Microbiol Infect 2015, 21:103.
19. Li JZ, et al. The size of the expressed HIV reservoir predicts timing of viral rebound after treatment interruption. AIDS. 2016 Jan 28;30(3):343-53.
20. Strategies for Management of Antiretroviral Therapy (SMART) Study Group, ElSadr WM, Lundgren JD, et al. CD4+ count-guided interruption of antiretroviral treatment. N Engl J Med. 2006 Nov 30;355(22):2283-96.
21. Tedaldi E, Peters L, Neuhaus J, et al. Opportunistic disease and mortality in patients coinfected with hepatitis B or C virus in the strategic management of antiretroviral therapy (SMART) study. Clin Infect Dis. 2008 Dec 1;47(11):1468- 75.
22. Monroe AK, Chander G, Moore RD. Control of medical comorbidities in individuals with HIV. J Acquir Immune Defic Syndr. 2011 Dec 15;58(5):458-62.

**APPENDIXES**

**APPENDIX 1**

# WMA Declaration of Helsinki - Ethical Principles for Medical Research Involving Human Subjects

Adopted by the 18th WMA General Assembly, Helsinki, Finland, June 1964
and amended by the:
29th WMA General Assembly, Tokyo, Japan, October 1975
35th WMA General Assembly, Venice, Italy, October 1983
41st WMA General Assembly, Hong Kong, September 1989
48th WMA General Assembly, Somerset West, Republic of South Africa, October 1996
52nd WMA General Assembly, Edinburgh, Scotland, October 2000 
53rd WMA General Assembly, Washington DC, USA, October 2002 (Note of Clarification added)
55th WMA General Assembly, Tokyo, Japan, October 2004 (Note of Clarification added)
59th WMA General Assembly, Seoul, Republic of Korea, October 2008
64th WMA General Assembly, Fortaleza, Brazil, October 2013

**Preamble**

1.         The World Medical Association (WMA) has developed the Declaration of Helsinki as a statement of ethical principles for medical research involving human subjects, including research on identifiable human material and data.

            The Declaration is intended to be read as a whole and each of its constituent paragraphs should be applied with consideration of all other relevant paragraphs.

2.         Consistent with the mandate of the WMA, the Declaration is addressed primarily to physicians. The WMA encourages others who are involved in medical research involving human subjects to adopt these principles.

**General Principles**

3.         The Declaration of Geneva of the WMA binds the physician with the words, “The health of my patient will be my first consideration,” and the International Code of Medical Ethics declares that, “A physician shall act in the patient's best interest when providing medical care.”

4.         It is the duty of the physician to promote and safeguard the health, well-being and rights of patients, including those who are involved in medical research. The physician's knowledge and conscience are dedicated to the fulfilment of this duty.

5.         Medical progress is based on research that ultimately must include studies involving human subjects.

6.         The primary purpose of medical research involving human subjects is to understand the causes, development and effects of diseases and improve preventive, diagnostic and therapeutic interventions (methods, procedures and treatments). Even the best proven interventions must be evaluated continually through research for their safety, effectiveness, efficiency, accessibility and quality.

7.         Medical research is subject to ethical standards that promote and ensure respect for all human subjects and protect their health and rights.

8.         While the primary purpose of medical research is to generate new knowledge, this goal can never take precedence over the rights and interests of individual research subjects.

9.         It is the duty of physicians who are involved in medical research to protect the life, health, dignity, integrity, right to self-determination, privacy, and confidentiality of personal information of research subjects. The responsibility for the protection of research subjects must always rest with the physician or other health care professionals and never with the research subjects, even though they have given consent.

10.       Physicians must consider the ethical, legal and regulatory norms and standards for research involving human subjects in their own countries as well as applicable international norms and standards. No national or international ethical, legal or regulatory requirement should reduce or eliminate any of the protections for research subjects set forth in this Declaration.

11.       Medical research should be conducted in a manner that minimises possible harm to the environment.

12.       Medical research involving human subjects must be conducted only by individuals with the appropriate ethics and scientific education, training and qualifications. Research on patients or healthy volunteers requires the supervision of a competent and appropriately qualified physician or other health care professional.

13.       Groups that are underrepresented in medical research should be provided appropriate access to participation in research.

14.       Physicians who combine medical research with medical care should involve their patients in research only to the extent that this is justified by its potential preventive, diagnostic or therapeutic value and if the physician has good reason to believe that participation in the research study will not adversely affect the health of the patients who serve as research subjects.

15.       Appropriate compensation and treatment for subjects who are harmed as a result of participating in research must be ensured.

**Risks, Burdens and Benefits**

16.       In medical practice and in medical research, most interventions involve risks and burdens.

           Medical research involving human subjects may only be conducted if the importance of the objective outweighs the risks and burdens to the research subjects.

17.       All medical research involving human subjects must be preceded by careful assessment of predictable risks and burdens to the individuals and groups involved in the research in comparison with foreseeable benefits to them and to other individuals or groups affected by the condition under investigation.

           Measures to minimise the risks must be implemented. The risks must be continuously monitored, assessed and documented by the researcher.

18.       Physicians may not be involved in a research study involving human subjects unless they are confident that the risks have been adequately assessed and can be satisfactorily managed.

           When the risks are found to outweigh the potential benefits or when there is conclusive proof of definitive outcomes, physicians must assess whether to continue, modify or immediately stop the study.

**Vulnerable Groups and Individuals**

19.       Some groups and individuals are particularly vulnerable and may have an increased likelihood of being wronged or of incurring additional harm.

           All vulnerable groups and individuals should receive specifically considered protection.

20.       Medical research with a vulnerable group is only justified if the research is responsive to the health needs or priorities of this group and the research cannot be carried out in a non-vulnerable group. In addition, this group should stand to benefit from the knowledge, practices or interventions that result from the research.

**Scientific Requirements and Research Protocols**

21.       Medical research involving human subjects must conform to generally accepted scientific principles, be based on a thorough knowledge of the scientific literature, other relevant sources of information, and adequate laboratory and, as appropriate, animal experimentation. The welfare of animals used for research must be respected.

22.       The design and performance of each research study involving human subjects must be clearly described and justified in a research protocol.

           The protocol should contain a statement of the ethical considerations involved and should indicate how the principles in this Declaration have been addressed. The protocol should include information regarding funding, sponsors, institutional affiliations, potential conflicts of interest, incentives for subjects and information regarding provisions for treating and/or compensating subjects who are harmed as a consequence of participation in the research study.

           In clinical trials, the protocol must also describe appropriate arrangements for post-trial provisions.

**Research Ethics Committees**

23.       The research protocol must be submitted for consideration, comment, guidance and approval to the concerned research ethics committee before the study begins. This committee must be transparent in its functioning, must be independent of the researcher, the sponsor and any other undue influence and must be duly qualified. It must take into consideration the laws and regulations of the country or countries in which the research is to be performed as well as applicable international norms and standards but these must not be allowed to reduce or eliminate any of the protections for research subjects set forth in this Declaration.

           The committee must have the right to monitor ongoing studies. The researcher must provide monitoring information to the committee, especially information about any serious adverse events. No amendment to the protocol may be made without consideration and approval by the committee. After the end of the study, the researchers must submit a final report to the committee containing a summary of the study’s findings and conclusions.

**Privacy and Confidentiality**

24.       Every precaution must be taken to protect the privacy of research subjects and the confidentiality of their personal information.

**Informed Consent**

25.       Participation by individuals capable of giving informed consent as subjects in medical research must be voluntary. Although it may be appropriate to consult family members or community leaders, no individual capable of giving informed consent may be enrolled in a research study unless he or she freely agrees.

26.       In medical research involving human subjects capable of giving informed consent, each potential subject must be adequately informed of the aims, methods, sources of funding, any possible conflicts of interest, institutional affiliations of the researcher, the anticipated benefits and potential risks of the study and the discomfort it may entail, post-study provisions and any other relevant aspects of the study. The potential subject must be informed of the right to refuse to participate in the study or to withdraw consent to participate at any time without reprisal. Special attention should be given to the specific information needs of individual potential subjects as well as to the methods used to deliver the information.

           After ensuring that the potential subject has understood the information, the physician or another appropriately qualified individual must then seek the potential subject’s freely-given informed consent, preferably in writing. If the consent cannot be expressed in writing, the non-written consent must be formally documented and witnessed.

           All medical research subjects should be given the option of being informed about the general outcome and results of the study.

27.       When seeking informed consent for participation in a research study the physician must be particularly cautious if the potential subject is in a dependent relationship with the physician or may consent under duress. In such situations the informed consent must be sought by an appropriately qualified individual who is completely independent of this relationship.

28.       For a potential research subject who is incapable of giving informed consent, the physician must seek informed consent from the legally authorised representative. These individuals must not be included in a research study that has no likelihood of benefit for them unless it is intended to promote the health of the group represented by the potential subject, the research cannot instead be performed with persons capable of providing informed consent, and the research entails only minimal risk and minimal burden.

29.       When a potential research subject who is deemed incapable of giving informed consent is able to give assent to decisions about participation in research, the physician must seek that assent in addition to the consent of the legally authorised representative. The potential subject’s dissent should be respected.

30.       Research involving subjects who are physically or mentally incapable of giving consent, for example, unconscious patients, may be done only if the physical or mental condition that prevents giving informed consent is a necessary characteristic of the research  group. In such circumstances the physician must seek informed consent from the legally authorised representative. If no such representative is available and if the research cannot be delayed, the study may proceed without informed consent provided that the specific reasons for involving subjects with a condition that renders them unable to give informed consent have been stated in the research protocol and the study has been approved by a research ethics committee. Consent to remain in the research must be obtained as soon as possible from the subject or a legally authorised representative.

31.       The physician must fully inform the patient which aspects of their care are related to the research. The refusal of a patient to participate in a study or the patient’s decision to withdraw from the study must never adversely affect the patient-physician relationship.

32.       For medical research using identifiable human material or data, such as research on material or data contained in biobanks or similar repositories, physicians must seek informed consent for its collection, storage and/or reuse. There may be exceptional situations where consent would be impossible or impracticable to obtain for such research. In such situations the research may be done only after consideration and approval of a research ethics committee.

**Use of Placebo**

33.       The benefits, risks, burdens and effectiveness of a new intervention must be tested against those of the best proven intervention(s), except in the following circumstances:

           Where no proven intervention exists, the use of placebo, or no intervention, is acceptable; or

           Where for compelling and scientifically sound methodological reasons the use of any intervention less effective than the best proven one, the use of placebo, or no intervention is necessary to determine the efficacy or safety of an intervention

           and the patients who receive any intervention less effective than the best proven one, placebo, or no intervention will not be subject to additional risks of serious or irreversible harm as a result of not receiving the best proven intervention.

           Extreme care must be taken to avoid abuse of this option.

**Post-Trial Provisions**

34.       In advance of a clinical trial, sponsors, researchers and host country governments should make provisions for post-trial access for all participants who still need an intervention identified as beneficial in the trial. This information must also be disclosed to participants during the informed consent process.

**Research Registration and Publication and Dissemination of Results**

35.       Every research study involving human subjects must be registered in a publicly accessible database before recruitment of the first subject.

36.       Researchers, authors, sponsors, editors and publishers all have ethical obligations with regard to the publication and dissemination of the results of research. Researchers have a duty to make publicly available the results of their research on human subjects and are accountable for the completeness and accuracy of their reports. All parties should adhere to accepted guidelines for ethical reporting. Negative and inconclusive as well as positive results must be published or otherwise made publicly available. Sources of funding, institutional affiliations and conflicts of interest must be declared in the publication. Reports of research not in accordance with the principles of this Declaration should not be accepted for publication.

**Unproven Interventions in Clinical Practice**

37.       In the treatment of an individual patient, where proven interventions do not exist or other known interventions have been ineffective, the physician, after seeking expert advice, with informed consent from the patient or a legally authorised representative, may use an unproven intervention if in the physician's judgement it offers hope of saving life, re-establishing health or alleviating suffering. This intervention should subsequently be made the object of research, designed to evaluate its safety and efficacy. In all cases, new information must be recorded and, where appropriate, made publicly available.

**APPENDIX 2**

**Pharmacovigilance**

In case of adverse events and serious adverse events during the study, the Promoter (Prof. Adriano Lazzarin) will be responsible of:

- Redaction of Register of adverse events and unexpected reactions; this one should be presented to Italian Minister if requested.
- Redaction and notification to Ethic Committee and to Italian Health Council no later than 15 days (7 days in case of lethal adverse event) all information about suspect serious adverse events and unexpected events.
- Annually presentation of all serious adverse events to Italian Health Council and to Ethical Committee.
- Communication to Ethical Committee of all deaths, also in case of progression of disease. A copy must be sent to Sanitary Direction and to the Pharmacy.

The Investigator will be responsible of:

- Communication of all SAEs to Study’s Promoter as soon as possible and, in any case, no later than 15 days (7 in case of fatal event) after the manifestation of the event. A copy of all SAEs should be reported to local Ethic Committee and to Pharmacy.

**APPENDIX 3**

**Toxicity Management**

**Grade 1 or 2 or 3 Toxicity**

Subjects who develop a Grade 1 or 2 AE or toxicity may continue the study.

Subjects who develop a Grade 3 AE or toxicity will be strictly monitored by the study physician to evaluate if it may be a transitory occurrence (it may return to Grade 2 or lower) or to decide for study discontinuation.

**Grade 4 Toxicity**

Subjects who develop a Grade 4 symptomatic AE or toxicity judged possibly related to study treatment status and requiring medical intervention will be discontinued pending complete medical evaluation of the event.

For subjects who experience a Grade 4 symptomatic AE or toxicity judged as not related to study treatment status or a grade 4 AEs for which no clear cause can be determined, the study physician will decide for a strict monitoring or for study discontinuation.

Subjects with Grade 4 asymptomatic laboratory abnormalities may continue the study if the investigator has evidence that the toxicity is not related to the study treatment status.

**APPENDIX 4**

**CLASSIFICATION SYSTEM FOR HIV INFECTION (CDC CLASSIFICATION)**

Reference - 1993 Revised Classification System for HIV Infection and Expanded Surveillance Case Definition for AIDS Among Adolescents and Adults

**Clinical Categories**

CD4+ T-Lymphocyte Categories

The three CD4+ T-lymphocyte categories are defined as follows:

- Category 1: greater than or equal to 500 cells/μL
- Category 2: 200-499 cells/μL
- Category 3: less than 200 cells/μL

The clinical categories of HIV infection are defined as follows:

**Category A**

Category A consists of one or more of the conditions listed below in an adolescent or adult (greater than or equal to 13 years) with documented HIV infection. Conditions listed in Categories B and C must not have occurred.

- Asymptomatic HIV infection
- Persistent generalized lymphadenopathy
- Acute (primary) HIV infection with accompanying illness or history of acute HIV infection

**Category B** (Symptomatic non-AIDS conditions)

Category B consists of symptomatic conditions in an HIV-infected adolescent or adult that are not included among conditions listed in clinical Category C and that meet at least one of the following criteria: a) the conditions are attributed to HIV infection or are indicative of a defect in cell-mediated immunity; or b) the conditions are considered by physicians to have a clinical course or to require management that is complicated by HIV infection. Examples of conditions in clinical Category B include, but are not limited to:

- Bacillary angiomatosis
- Candidiasis, oropharyngeal (thrush)
- Candidiasis, vulvovaginal; persistent, frequent, or poorly responsive to therapy
- Cervical dysplasia (moderate or severe)/cervical carcinoma in situ
- Constitutional symptoms, such as fever (38.5 C) or diarrhoea lasting greater than 1 month
- Hairy leukoplakia, oral
- Herpes zoster (shingles), involving at least two distinct episodes or more than one dermatome
- Idiopathic thrombocytopenic purpura
- Listeriosis
- Pelvic inflammatory disease, particularly if complicated by tubo-ovarian abscess
- Peripheral neuropathy

For classification purposes, Category B conditions take precedence over those in Category A. For example, someone previously treated for oral or persistent vaginal candidiasis (and who has not developed a Category C disease) but who is now asymptomatic should be classified in clinical Category B.

**Category C** (AIDS indicator conditions as defined by diagnostic or presumptive)

Category C includes the clinical conditions listed in the AIDS surveillance case definition. For classification purposes, once a Category C condition has occurred, the person will remain in Category C.

**LISTING OF AIDS-DEFINING ILLNESSES**

(Based on the CDC’s 1993 Revised Classification System for HIV Infection and Expanded Surveillance Case Definition for AIDS among Adolescents and Adult)

- Candidiasis of bronchi, trachea, or lungs
- Candidiasis, esophageal
- Cervical cancer, invasive *
- Coccidioidomycosis, disseminated or extrapulmonary
- Cryptococcosis, extrapulmonary
- Cryptosporidiosis, chronic intestinal (greater than 1 month's duration)
- Cytomegalovirus disease (other than liver, spleen, or nodes)
- Cytomegalovirus retinitis (with loss of vision)
- Encephalopathy, HIV-related
- Herpes simplex: chronic ulcer(s) (greater than 1 month's duration); or bronchitis, pneumonitis, or esophagitis
- Histoplasmosis, disseminated or extrapulmonary
- Isosporiasis, chronic intestinal (greater than 1 month's duration)
- Kaposi's sarcoma
- Lymphoma, Burkitt's (or equivalent term)
- Lymphoma, immunoblastic (or equivalent term)
- Lymphoma, primary, of brain
- Mycobacterium avium complex or M. kansasii, disseminated or extrapulmonary
- Mycobacterium tuberculosis, any site (pulmonary * or extrapulmonary)
- Mycobacterium, other species or unidentified species, disseminated or extrapulmonary
- Pneumocystis carinii pneumonia
- Pneumonia, recurrent *
- Progressive multifocal leukoencephalopathy
- Salmonella septicemia, recurrent
- Toxoplasmosis of brain
- Wasting syndrome due to HIV

**Note**: although CD4+<200 cells/µL is considered an AIDS-defining condition per CDC guidelines, it will be adjudicated ans will not require immediate notification since it is based on laboratory results only.

**APPENDIX 5**

**GUIDELINES FOR DIAGNOSIS OF AIDS- DEFINING CONDITIONS**

**Cryptosporidiosis, Isosporiasis, Kaposi's sarcoma, Lymphoma, Pneumocystis carinii pneumonia, Progressive multifocal leukoencephalopathy, Toxoplasmosis, Cervical cancer**: Microscopy (histology or cytology)

**Candidiasis**: Gross inspection by endoscopy or autopsy or by microscopy (histology or cytology) on a specimen obtained directly from the tissues affected (including scrapings from the mucosal surface), not from a culture

**Coccidioidomycosis, Cryptococcosis, Cytomegalovirus, Herpes simplex virus, Histoplasmosis**: microscopy (histology or cytology), culture, or detection of antigen in a specimen obtained directly from the tissues affected or a fluid from those tissues

**Tuberculosis, Other mycobacteriosis, Salmonellosis:** Culture

**HIV encephalopathy (dementia):** clinical findings of disabling cognitive or motor dysfunction interfering with occupation or activities of daily living, progressing over weeks to months, in the absence of a concurrent illness or condition other than HIV infection that could explain the findings. Methods to rule out such concurrent illness and conditions must include cerebrospinal fluid examination and either brain imaging (computed tomography or magnetic resonance) or autopsy.

**HIV wasting syndrome**: findings of profound involuntary weight loss of greater than 10% of baseline body weight plus either chronic diarrhoea (at least two loose stools per day for greater than or equal to 30 days), or chronic weakness and documented fever (for greater than or equal to 30 days, intermittent or constant) in the absence of a concurrent illness or condition other than HIV infection that could explain the findings (e.g., cancer, tuberculosis, cryptosporidiosis, or other specific enteritis).

**Pneumonia, recurrent:** recurrent (more than one episode in a 1-year period), acute (new x-ray evidence not present earlier) pneumonia diagnosed by both: a) culture (or other organism-specific diagnostic method) obtained from a clinically reliable specimen of a pathogen that typically causes pneumonia (other than Pneumocystis carinii or Mycobacterium tuberculosis), and b) radiological evidence of pneumonia; cases that do not have laboratory confirmation of a causative organism for one of the episodes of pneumonia will be considered to be presumptively diagnosed.

**Suggested guidelines for presumptive diagnosis of diseases indicative of AIDS**

**Candidiasis of the esophagus**:

1. Recent onset of retrosternal pain on swallowing; AND
2. Oral candidiasis diagnosed by the gross appearance of white patches or plaques on an erythematous base or by the microscopic appearance of fungal mycelial filaments from a noncultured specimen scraped from the oral mucosa.

**Cytomegalovirus retinitis:** a characteristic appearance on serial ophthalmoscopic examinations (e.g., discrete patches of retinal whitening with distinct borders, spreading in a centrifugal manner along the paths of blood vessels, progressing over several months, and frequently associated with retinal vasculitis, haemorrhage, and necrosis). Resolution of active disease leaves retinal scarring and atrophy with retinal pigment epithelial mottling.

**Mycobacteriosis**: microscopy of a specimen from stool or normally sterile body fluids or tissue from a site other than lungs, skin, or cervical or hilar lymph nodes that shows acid-fast bacilli of a species not identified by culture.

**Kaposi's sarcoma**: a characteristic gross appearance of an erythematous or violaceous plaque-like lesion on skin or mucous membrane. (Note: Presumptive diagnosis of Kaposi's sarcoma should not be made by clinicians who have seen few cases of it.)

**Pneumocystis carinii pneumonia** :

1. A history of dyspnoea on exertion or non-productive cough of recent onset (within the past 3 months); AND
2. Chest x-ray evidence of diffuse bilateral interstitial infiltrates or evidence by gallium scan of diffuse bilateral pulmonary disease; AND
3. Arterial blood gas analysis showing an arterial pO_2_ of less than 70 mm Hg or a low respiratory diffusing capacity (less than 80% of predicted values) or an increase in the alveolar-arterial oxygen tension gradient; AND
4. No evidence of a bacterial pneumonia.

**Pneumonia, recurrent**: recurrent (more than one episode in a 1-year period), acute (new symptoms, signs, or x-ray evidence not present earlier) pneumonia diagnosed on clinical or radiological grounds by the patient's physician.

**Toxoplasmosis of brain**:

1. Recent onset of a focal neurological abnormality consistent with intracranial disease or a reduced level of consciousness; AND
2. Evidence by brain imaging (computed tomography or nuclear magnetic resonance) of a lesion having a mass effect or the radiographic appearance of which is enhanced by injection of contrast medium; AND
3. Serum antibody to toxoplasmosis or successful response to therapy for toxoplasmosis.

**Tuberculosis, pulmonary**: when bacteriologic confirmation is not available, other reports may be considered to be verified cases of pulmonary tuberculosis if the criteria of the Division of Tuberculosis Elimination, National Center for Prevention Services, CDC, are used.

**APPENDIX 6**

**Division of AIDS (DAIDS) Table for Grading the Severity of Adult and Pediatric Adverse Events**

**Version 2.0 - November 2014**

**Instructions for Use**

**General Considerations**

The *Division of AIDS* (*DAIDS) Table for Grading the Severity of Adult and Pediatric Adverse Events, Version 2.0* consists of parameters, or AEs, with severity grading guidance that are to be used in DAIDS clinical trials for safety data reporting to maintain accuracy and consistency in the evaluation of AEs. The term “severe” is not the same as the term “serious” in classifying AEs. The severity of a specific event describes its intensity, and it is the intensity which is graded. Seriousness, which is not graded, relates to an outcome of an AE and is a regulatory definition.

Clinical sites are encouraged to report parameters in the DAIDS AE grading table as they are written to maintain data consistency across clinical trials. However, since some parameters can be reported with more specificity, clinical sites are encouraged to report parameters that convey additional clinical information. For example, diarrhea could be reported as neonatal diarrhea; seizures, as febrile seizures; and pain, as jaw pain.

The DAIDS AE grading table provides an AE severity grading scale ranging from grades 1 to 5 with descriptions for each AE based on the following general guidelines:

• Grade 1 indicates a mild event

• Grade 2 indicates a moderate event

• Grade 3 indicates a severe event

• Grade 4 indicates a potentially life-threatening event

• Grade 5 indicates death (*Note*: This grade is not specifically listed on each page of the grading table).

Other points to consider include:

• Use parameters defined by age and sex values as applicable.

• Male and female sex are defined as sex at birth.

• Unless noted, laboratory values are for term neonates. Preterm neonates should be assessed using local laboratory normal ranges.

• Where applicable, Standard International (SI) units are included in italics.

**Selecting and Reporting a Primary AE Term**

When selecting a primary AE term to report, sites should select the term that best describes what occurred to the participant. For example, a participant may present with itching, urticaria, flushing, angioedema of the face, and dyspnea. If the underlying diagnosis is determined to be an acute allergic reaction, sites should report “Acute Allergic Reaction” as the primary AE term.

Primary AE terms should be reported using the DAIDS Adverse Experience Reporting System (DAERS) only if they meet expedited reporting criteria. However, all primary AE terms should be reported using protocol-specific case report forms (CRFs). Because the reported information is stored in different databases (i.e., safety and clinical), sites should report primary AE terms using the same terminology for data consistency.

When reporting using DAERS, other clinically significant events associated with a primary AE term that more fully describe the nature, severity, or complications of the primary AE term should be entered in the “Other Events” section. However, the severity grade for these events must be lower than or equal to the severity grade of the primary AE term. In the example above, dyspnea and angioedema of the face may be entered in the “Other Events” section, because they are more descriptive and provide additional information on the severity of the acute allergic reaction. However, their severity grades must be lower than or equal to the severity grade of the primary AE term of “Acute Allergic Reaction”.

Differences exist in the reporting and recording of information (e.g., signs and symptoms, clinically significant events) in DAERS and CRFs. Therefore, sites should refer to their protocols and CRF requirements for further instructions.

**Grading Adult and Pediatric AEs**

When a single parameter is not appropriate for grading an AE in both adult and pediatric populations, separate parameters with specified age ranges are provided. If no distinction

between adult and pediatric populations has been made, the listed parameter should be used for grading an AE in both populations.

**Reporting Pregnancy Outcomes**

In the *Pregnancy, Puerperium, and Perinatal* section, all parameters are pregnancy outcomes and should be reported using the mother's participant ID. If an infant is not enrolled in the same study as the mother, any identified birth defects should be reported using the mother's participant ID. However, if an infant is enrolled in the same study as the mother or in another study, any identified birth defects should be reported using the infant's participant ID. Sites should refer to the applicable network standards for reporting abnormal pregnancy outcomes on the CRFs.

**Determining Severity Grade for Parameters between Grades**

If the severity of an AE could fall in either one of two grades (i.e., the severity of an AE could be either grade 2 or grade 3), sites should select the higher of the two grades.

**Laboratory Values**

*General.* An asymptomatic, abnormal laboratory finding without an accompanying AE should not be reported to DAIDS in an expedited timeframe unless it meets protocol-specific reporting requirements. Sites should refer to the applicable network standards for reporting abnormal laboratory findings on the CRFs.

*Values below Grade 1.* Any laboratory value that is between the ULN and grade 1 (for high values) or the LLN and grade 1 (for low values) should not be graded or reported as an AE. Sites should consult the *Manual for Expedited Reporting of Adverse Events to DAIDS, Version 2.0* and their protocol when making an assessment of the need to report an AE.

*Overlap of Local Laboratory Normal Values with Grading Table Ranges*. When local laboratory normal values fall within grading table laboratory ranges, the severity grading is based on the ranges in the grading table unless there is a protocol-specific grading criterion for the laboratory value. For example, “Magnesium, Low" has a grade 1 range of 1.2 to < 1.4 mEq/L, while a particular laboratory’s normal range for magnesium may be 1.3 to 2.8 mEq/L. If a study participant’s magnesium laboratory value is 1.3 mEq/L, the laboratory value should be graded as grade 1.

**Appendix Usage**

Appendix A takes priority over the main grading table in all assessments of total bilirubin for term and preterm neonates.

**Using Addenda 1-3: Grading Tables Used in Microbicide Studies**

In protocols involving topical application of products to the female and male genital tracts or rectum, strong consideration should be given to using Addenda 1-3 (see below) as the primary grading tables for these areas. Although these grading tables are used specifically in microbicide studies, they may be used in other protocols as adjuncts to the main grading table (i.e., the *Division of AIDS (AIDS) Table for Grading the Severity of Adult and Pediatric Adverse Events, Version 2.0*). It should be clearly stated in a protocol which addendum is being used as the primary grading table (and thus takes precedence over the main grading table) and which addendum is being used in a complementary fashion.

• Addendum 1 – Female Genital Grading Table for Use in Microbicide Studies – PDF

• Addendum 2 – Male Genital Grading Table for Use in Microbicide Studies – PDF

• Addendum 3 – Rectal Grading Table for Use in Microbicide Studies – PDF

**Estimating Severity Grade for Parameters Not Identified in the Grading Table**

The functional table below should be used to grade the severity of an AE that is not specifically identified in the grading table. In addition, all deaths related to an AE are to be classified as grade 5.

| **PARAMETER** | **GRADE 1 MILD** | **GRADE 2 MODERATE** | **GRADE 3 SEVERE** | **GRADE 4**  **POTENTIALLY LIFE-THREATENING** |
| --- | --- | --- | --- | --- |
| **Clinical** adverse event **NOT** identified elsewhere in the grading table | Mild symptoms causing no or minimal interference with usual social & functional activities with intervention not indicated | Moderate symptoms causing greater than minimal interference with usual social & functional activities with intervention indicated | Severe symptoms causing inability to perform usual social & functional activities with intervention or hospitalization indicated | Potentially life-threatening symptoms causing inability to perform basic self-care functions with intervention indicated to prevent permanent impairment, persistent disability, or death |

*Major Clinical Conditions Cardiovascular*

| **PARAMETER** | **GRADE 1 MILD** | **GRADE 2 MODERATE** | **GRADE 3 SEVERE** | **GRADE 4 POTENTIALLY LIFE-THREATENING** |
| --- | --- | --- | --- | --- |
| **Arrhythmia** (by ECG or physical examination)  *Specify type, if applicable* | No symptoms AND No intervention indicated | No symptoms AND Non-urgent intervention indicated | Non-life-threatening symptoms AND Non-urgent intervention indicated | Life-threatening arrhythmia OR Urgent intervention indicated |
| **Blood Pressure Abnormalities^1^**  ***Hypertension*** *(with the lowest reading taken after repeat testing during a visit)*  *≥ 18 years of age* | 140 to < 160 mmHg systolic OR 90 to < 100 mmHg diastolic | ≥ 160 to < 180 mmHg systolic OR ≥ 100 to < 110 mmHg diastolic | ≥ 180 mmHg systolic OR ≥ 110 mmHg diastolic | Life-threatening consequences in a participant not previously diagnosed with hypertension (e.g., malignant hypertension) OR Hospitalization indicated |
| *< 18 years of age* | > 120/80 mmHg | ≥ 95th to < 99th percentile + 5 mmHg adjusted for age, height, and gender (systolic and/or diastolic) | ≥ 99th percentile + 5 mmHg adjusted for age, height, and gender (systolic and/or diastolic) | Life-threatening consequences in a participant not previously diagnosed with hypertension (e.g., malignant hypertension) OR Hospitalization indicated |
| ***Hypotension*** | No symptoms | Symptoms corrected with oral fluid replacement | Symptoms AND IV fluids indicated | Shock requiring use of vasopressors or mechanical assistance to maintain blood pressure |
| **Cardiac Ischemia or Infarction**  *Report only one* | NA | NA | New symptoms with ischemia (stable angina) OR New testing consistent with ischemia | Unstable angina OR Acute myocardial infarction |
| **Heart Failure** | No symptoms AND Laboratory or cardiac imaging abnormalities | Symptoms with mild to moderate activity or exertion | Symptoms at rest or with minimal activity or exertion (e.g., hypoxemia) OR Intervention indicated (e.g., oxygen) | Life-threatening consequences OR Urgent intervention indicated (e.g., vasoactive medications, ventricular assist device, heart transplant) |
| **Hemorrhage** (with significant acute blood loss) | NA | Symptoms AND No transfusion indicated | Symptoms AND Transfusion of ≤ 2 units packed RBCs indicated | Life-threatening hypotension OR Transfusion of > 2 units packed RBCs (for children, packed RBCs > 10 cc/kg) indicated |

1 Blood pressure norms for children < 18 years of age can be found in: Expert Panel on Integrated Guidelines for Cardiovascular Health and Risk Reduction in Children and Adolescents. Pediatrics 2011;128;S213; originally published online November 14, 2011; DOI: 10.1542/peds.2009-2107C.

*Cardiovascular*

| **PARAMETER** | **GRADE 1 MILD** | **GRADE 2 MODERATE** | **GRADE 3 SEVERE** | **GRADE 4 POTENTIALLY LIFE-THREATENING** |
| --- | --- | --- | --- | --- |
| **Prolonged PR Interval or AV Block**  *Report only one*  *> 16 years of age* | PR interval 0.21 to < 0.25 seconds | PR interval ≥ 0.25 seconds OR Type I 2nd degree AV block | Type II 2nd degree AV block OR Ventricular pause ≥ 3.0 seconds | Complete AV block |
| *≤ 16 years of age* | 1st degree AV block (PR interval > normal for age and rate) | Type I 2nd degree AV block | Type II 2nd degree AV block OR Ventricular pause ≥ 3.0 seconds | Complete AV block |
| **Prolonged QTc Interval^2^** | 0.45 to 0.47 seconds | > 0.47 to 0.50 seconds | > 0.50 seconds OR ≥ 0.06 seconds above baseline | Life-threatening consequences (e.g., Torsade de pointes, other associated serious ventricular dysrhythmia) |
| **Thrombosis or Embolism**  *Report only one* | NA | Symptoms AND No intervention indicated | Symptoms AND Intervention indicated | Life-threatening embolic event (e.g., pulmonary embolism, thrombus) |

2 As per Bazett’s formula.

*Dermatologic*

| **PARAMETER** | **GRADE 1 MILD** | **GRADE 2 MODERATE** | **GRADE 3 SEVERE** | **GRADE 4 POTENTIALLY LIFE-THREATENING** |
| --- | --- | --- | --- | --- |
| **Alopecia** (scalp only) | Detectable by study participant, caregiver, or physician AND Causing no or minimal interference with usual social & functional activities | Obvious on visual inspection AND Causing greater than minimal interference with usual social & functional activities | NA | NA |
| **Bruising** | Localized to one area | Localized to more than one area | Generalized | NA |
| **Cellulitis** | NA | Non-parenteral treatment indicated (e.g., oral antibiotics, antifungals, antivirals) | IV treatment indicated (e.g., IV antibiotics, antifungals, antivirals) | Life-threatening consequences (e.g., sepsis, tissue necrosis) |
| **Hyperpigmentation** | Slight or localized causing no or minimal interference with usual social & functional activities | Marked or generalized causing greater than minimal interference with usual social & functional activities | NA | NA |
| **Hypopigmentation** | Slight or localized causing no or minimal interference with usual social & functional activities | Marked or generalized causing greater than minimal interference with usual social & functional activities | NA | NA |
| **Petechiae** | Localized to one area | Localized to more than one area | Generalized | NA |
| **Pruritus^3^** (without skin lesions) | Itching causing no or minimal interference with usual social & functional activities | Itching causing greater than minimal interference with usual social & functional activities | Itching causing inability to perform usual social & functional activities | NA |
| **Rash**  *Specify type, if applicable* | Localized rash | Diffuse rash OR Target lesions | Diffuse rash AND Vesicles or limited number of bullae or superficial ulcerations of mucous membrane limited to one site | Extensive or generalized bullous lesions OR Ulceration of mucous membrane involving two or more distinct mucosal sites OR Stevens-Johnson syndrome OR Toxic epidermal necrolysis |

3 For pruritus associated with injections or infusions, see the Site Reactions to Injections and Infusions section (page 23).

Endocrine and Metabolic

| **PARAMETER** | **GRADE 1 MILD** | **GRADE 2 MODERATE** | **GRADE 3 SEVERE** | **GRADE 4 POTENTIALLY LIFE-THREATENING** |
| --- | --- | --- | --- | --- |
| **Diabetes Mellitus** | Controlled without medication | Controlled with medication OR Modification of current medication regimen | Uncontrolled despite treatment modification OR Hospitalization for immediate glucose control indicated | Life-threatening consequences (e.g., ketoacidosis, hyperosmolar non-ketotic coma, end organ failure) |
| **Gynecomastia** | Detectable by study participant, caregiver, or physician AND Causing no or minimal interference with usual social & functional activities | Obvious on visual inspection AND Causing pain with greater than minimal interference with usual social & functional activities | Disfiguring changes AND Symptoms requiring intervention or causing inability to perform usual social & functional activities | NA |
| **Hyperthyroidism** | No symptoms AND Abnormal laboratory value | Symptoms causing greater than minimal interference with usual social & functional activities OR Thyroid suppression therapy indicated | Symptoms causing inability to perform usual social & functional activities OR Uncontrolled despite treatment modification | Life-threatening consequences (e.g., thyroid storm) |
| **Hypothyroidism** | No symptoms AND Abnormal laboratory value | Symptoms causing greater than minimal interference with usual social & functional activities OR Thyroid replacement therapy indicated | Symptoms causing inability to perform usual social & functional activities OR Uncontrolled despite treatment modification | Life-threatening consequences (e.g., myxedema coma) |
| **Lipoatrophy^4^** | Detectable by study participant, caregiver, or physician AND Causing no or minimal interference with usual social & functional activities | Obvious on visual inspection AND Causing greater than minimal interference with usual social & functional activities | Disfiguring changes | NA |
| **Lipohypertrophy^5^** | Detectable by study participant, caregiver, or physician AND Causing no or minimal interference with usual social & functional activities | Obvious on visual inspection AND Causing greater than minimal interference with usual social & functional activities | Disfiguring changes | NA |

4 Definition: A disorder characterized by fat loss in the face, extremities, and buttocks.

5 Definition: A disorder characterized by abnormal fat accumulation on the back of the neck, breasts, and abdomen.

*Gastrointestinal*

| **PARAMETER** | **GRADE 1 MILD** | **GRADE 2 MODERATE** | **GRADE 3 SEVERE** | **GRADE 4 POTENTIALLY LIFE-THREATENING** |
| --- | --- | --- | --- | --- |
| **Anorexia** | Loss of appetite without decreased oral intake | Loss of appetite associated with decreased oral intake without significant weight loss | Loss of appetite associated with significant weight loss | Life-threatening consequences OR Aggressive intervention indicated (e.g., tube feeding, total parenteral nutrition) |
| **Ascites** | No symptoms | Symptoms AND Intervention indicated (e.g., diuretics, therapeutic paracentesis) | Symptoms recur or persist despite intervention | Life-threatening consequences |
| **Bloating or Distension**  *Report only one* | Symptoms causing no or minimal interference with usual social & functional activities | Symptoms causing greater than minimal interference with usual social & functional activities | Symptoms causing inability to perform usual social & functional activities | NA |
| **Cholecystitis** | NA | Symptoms AND Medical intervention indicated | Radiologic, endoscopic, or operative intervention indicated | Life-threatening consequences (e.g., sepsis, perforation) |
| **Constipation** | NA | Persistent constipation requiring regular use of dietary modifications, laxatives, or enemas | Obstipation with manual evacuation indicated | Life-threatening consequences (e.g., obstruction) |
| **Diarrhea** *≥ 1 year of age* | Transient or intermittent episodes of unformed stools OR Increase of ≤ 3 stools over baseline per 24-hour period | Persistent episodes of unformed to watery stools OR Increase of 4 to 6 stools over baseline per 24-hour period | Increase of ≥ 7 stools per 24-hour period OR IV fluid replacement indicated | Life-threatening consequences (e.g., hypotensive shock) |
| *< 1 year of age* | Liquid stools (more unformed than usual) but usual number of stools | Liquid stools with increased number of stools OR Mild dehydration | Liquid stools with moderate dehydration | Life-threatening consequences (e.g., liquid stools resulting in severe dehydration, hypotensive shock) |
| **Dysphagia or Odynophagia**  *Report only one and specify location* | Symptoms but able to eat usual diet | Symptoms causing altered dietary intake with no intervention indicated | Symptoms causing severely altered dietary intake with intervention indicated | Life-threatening reduction in oral intake |
| **Gastrointestinal Bleeding** | Not requiring intervention other than iron supplement | Endoscopic intervention indicated | Transfusion indicated | Life-threatening consequences (e.g., hypotensive shock) |
| **Mucositis or Stomatitis**  **Report only one and specify location** | Mucosal erythema | Patchy pseudomembranes or ulcerations | Confluent pseudomembranes or ulcerations OR Mucosal bleeding with minor trauma | Life-threatening consequences (e.g., aspiration, choking) OR Tissue necrosis OR Diffuse spontaneous mucosal bleeding |
| **Nausea** | Transient (< 24 hours) or intermittent AND No or minimal interference with oral intake | Persistent nausea resulting in decreased oral intake for 24 to 48 hours | Persistent nausea resulting in minimal oral intake for > 48 hours OR Rehydration indicated (e.g., IV fluids) | Life-threatening consequences (e.g., hypotensive shock) |
| **Pancreatitis** | NA | Symptoms with hospitalization not indicated | Symptoms with hospitalization indicated | Life-threatening consequences (e.g., circulatory failure, hemorrhage, sepsis) |
| **Perforation**  **(colon or rectum)** | NA | NA | Intervention indicated | Life-threatening consequences |
| **Proctitis** | Rectal discomfort with no intervention indicated | Symptoms causing greater than minimal interference with usual social & functional activities OR Medical intervention indicated | Symptoms causing inability to perform usual social & functional activities OR Operative intervention indicated | Life-threatening consequences (e.g., perforation) |
| **Rectal Discharge** | Visible discharge | Discharge requiring the use of pads | NA | NA |
| **Vomiting** | Transient or intermittent AND No or minimal interference with oral intake | Frequent episodes with no or mild dehydration | Persistent vomiting resulting in orthostatic hypotension OR Aggressive rehydration indicated (e.g., IV fluids) | Life-threatening consequences (e.g., hypotensive shock) |

*Musculoskeletal*

| **PARAMETER** | **GRADE 1 MILD** | **GRADE 2 MODERATE** | **GRADE 3 SEVERE** | **GRADE 4 POTENTIALLY LIFE-THREATENING** |
| --- | --- | --- | --- | --- |
| **Arthralgia** | Joint pain causing no or minimal interference with usual social & functional activities | Joint pain causing greater than minimal interference with usual social & functional activities | Joint pain causing inability to perform usual social & functional activities | Disabling joint pain causing inability to perform basic self-care functions |
| **Arthritis** | Stiffness or joint swelling causing no or minimal interference with usual social & functional activities | Stiffness or joint swelling causing greater than minimal interference with usual social & functional activities | Stiffness or joint swelling causing inability to perform usual social & functional activities | Disabling joint stiffness or swelling causing inability to perform basic self-care functions |
| **Myalgia** (generalized) | Muscle pain causing no or minimal interference with usual social & functional activities | Muscle pain causing greater than minimal interference with usual social & functional activities | Muscle pain causing inability to perform usual social & functional activities | Disabling muscle pain causing inability to perform basic self-care functions |
| **Osteonecrosis** | NA | No symptoms but with radiographic findings AND No operative intervention indicated | Bone pain with radiographic findings OR Operative intervention indicated | Disabling bone pain with radiographic findings causing inability to perform basic self-care functions |
| **Osteopenia^6^** *≥ 30 years of age* | BMD t-score -2.5 to -1 | NA | NA | NA |
| *< 30 years of age* | BMD z-score -2 to -1 | NA | NA | NA |
| **Osteoporosis^6^** *≥ 30 years of age* | NA | BMD t-score < -2.5 | Pathologic fracture (e.g., compression fracture causing loss of vertebral height) | Pathologic fracture causing life-threatening consequences |
| *< 30 years of age* | NA | BMD z-score < -2 | Pathologic fracture (e.g., compression fracture causing loss of vertebral height) | Pathologic fracture causing life-threatening consequences |

6 BMD t and z scores can be found in: Kanis JA on behalf of the World Health Organization Scientific Group (2007). Assessment of osteoporosis at the primary health-care level. Technical Report. World Health Organization Collaborating Centre for Metabolic Bone Diseases, University of Sheffield, UK. 2007: Printed by the University of Sheffield.

*Neurologic*

| **PARAMETER** | **GRADE 1 MILD** | **GRADE 2 MODERATE** | **GRADE 3 SEVERE** | **GRADE 4 POTENTIALLY LIFE-THREATENING** |
| --- | --- | --- | --- | --- |
| **Acute CNS Ischemia** | NA | NA | Transient ischemic attack | Cerebral vascular accident (e.g., stroke with neurological deficit) |
| **Altered Mental Status** (for Dementia, see *Cognitive, Behavioral, or Attentional Disturbance* below) | Changes causing no or minimal interference with usual social & functional activities | Mild lethargy or somnolence causing greater than minimal interference with usual social & functional activities | Confusion, memory impairment, lethargy, or somnolence causing inability to perform usual social & functional activities | Delirium OR Obtundation OR Coma |
| **Ataxia** | Symptoms causing no or minimal interference with usual social & functional activities OR No symptoms with ataxia detected on examination | Symptoms causing greater than minimal interference with usual social & functional activities | Symptoms causing inability to perform usual social & functional activities | Disabling symptoms causing inability to perform basic self-care functions |
| **Cognitive, Behavioral, or Attentional Disturbance** (includes dementia and attention deficit disorder)  *Specify type, if applicable* | Disability causing no or minimal interference with usual social & functional activities OR Specialized resources not indicated | Disability causing greater than minimal interference with usual social & functional activities OR Specialized resources on part-time basis indicated | Disability causing inability to perform usual social & functional activities OR Specialized resources on a full-time basis indicated | Disability causing inability to perform basic self-care functions OR Institutionalization indicated |
| **Developmental Delay** *< 18 years of age*  *Specify type, if applicable* | Mild developmental delay, either motor or cognitive, as determined by comparison with a developmental screening tool appropriate for the setting | Moderate developmental delay, either motor or cognitive, as determined by comparison with a developmental screening tool appropriate for the setting | Severe developmental delay, either motor or cognitive, as determined by comparison with a developmental screening tool appropriate for the setting | Developmental regression, either motor or cognitive, as determined by comparison with a developmental screening tool appropriate for the setting |
| **Headache** | Symptoms causing no or minimal interference with usual social & functional activities | Symptoms causing greater than minimal interference with usual social & functional activities | Symptoms causing inability to perform usual social & functional activities | Symptoms causing inability to perform basic self-care functions OR Hospitalization indicated OR Headache with significant impairment of alertness or other neurologic function |
| **Neuromuscular Weakness (includes myopathy and neuropathy)**  **Specify type, if applicable** | Minimal muscle weakness causing no or minimal interference with usual social & functional activities OR No symptoms with decreased strength on examination | Muscle weakness causing greater than minimal interference with usual social & functional activities | Muscle weakness causing inability to perform usual social & functional activities | Disabling muscle weakness causing inability to perform basic self-care functions OR Respiratory muscle weakness impairing ventilation |
| **Neurosensory Alteration (includes paresthesia and painful neuropathy)**  **Specify type, if applicable** | Minimal paresthesia causing no or minimal interference with usual social & functional activities OR No symptoms with sensory alteration on examination | Sensory alteration or paresthesia causing greater than minimal interference with usual social & functional activities | Sensory alteration or paresthesia causing inability to perform usual social & functional activities | Disabling sensory alteration or paresthesia causing inability to perform basic self-care functions |
| **Seizures New Onset Seizure**  **≥ 18 years of age** | NA | NA | 1 to 3 seizures | Prolonged and repetitive seizures (e.g., status epilepticus) OR Difficult to control (e.g., refractory epilepsy) |
| **< 18 years of age**  **(includes new or pre-existing febrile seizures)** | Seizure lasting < 5 minutes with < 24 hours postictal state | Seizure lasting 5 to < 20 minutes with < 24 hours postictal state | Seizure lasting ≥ 20 minutes OR > 24 hours postictal state | Prolonged and repetitive seizures (e.g., status epilepticus) OR Difficult to control (e.g., refractory epilepsy) |
| **Pre-existing Seizure** | NA | Increased frequency from previous level of control without change in seizure character | Change in seizure character either in duration or quality (e.g., severity or focality) | Prolonged and repetitive seizures (e.g., status epilepticus) OR Difficult to control (e.g., refractory epilepsy) |
| **Syncope** | Near syncope without loss of consciousness (e.g., pre-syncope) | Loss of consciousness with no intervention indicated | Loss of consciousness AND Hospitalization or intervention required | NA |

*Pregnancy, Puerperium, and Perinatal*

| **PARAMETER** | **GRADE 1 MILD** | **GRADE 2 MODERATE** | **GRADE 3 SEVERE** | **GRADE 4 POTENTIALLY LIFE-THREATENING** |
| --- | --- | --- | --- | --- |
| **Fetal Death or Stillbirth** (report using mother’s participant ID)  *Report only one* | NA | NA | Fetal loss occurring at ≥ 20 weeks gestation | NA |
| **Preterm Delivery^7^** (report using mother’s participant ID) | Delivery at 34 to < 37 weeks gestational age | Delivery at 28 to < 34 weeks gestational age | Delivery at 24 to < 28 weeks gestational age | Delivery at < 24 weeks gestational age |
| **Spontaneous Abortion or Miscarriage^8^** (report using mother’s participant ID)  *Report only one* | Chemical pregnancy | Uncomplicated spontaneous abortion or miscarriage | Complicated spontaneous abortion or miscarriage | NA |

7 Definition: A delivery of a live-born neonate occurring at ≥ 20 to < 37 weeks gestational age.

8 Definition: A clinically recognized pregnancy occurring at < 20 weeks gestational age.

*Psychiatric*

| **PARAMETER** | **GRADE 1 MILD** | **GRADE 2 MODERATE** | **GRADE 3 SEVERE** | **GRADE 4 POTENTIALLY LIFE-THREATENING** |
| --- | --- | --- | --- | --- |
| **Insomnia** | Mild difficulty falling asleep, staying asleep, or waking up early | Moderate difficulty falling asleep, staying asleep, or waking up early | Severe difficulty falling asleep, staying asleep, or waking up early | NA |
| **Psychiatric Disorders**  (includes anxiety,  depression, mania, and  psychosis)  *Specify disorder* | Symptoms with intervention not indicated OR Behavior causing no or minimal interference with usual social & functional activities | Symptoms with intervention indicated OR Behavior causing greater than minimal interference with usual social & functional activities | Symptoms with hospitalization indicated OR Behavior causing inability to perform usual social & functional activities | Threatens harm to self or others OR Acute psychosis OR Behavior causing inability to perform basic self-care functions |
| **Suicidal Ideation or Attempt**  *Report only one* | Preoccupied with thoughts of death AND No wish to kill oneself | Preoccupied with thoughts of death AND Wish to kill oneself with no specific plan or intent | Thoughts of killing oneself with partial or complete plans but no attempt to do so OR Hospitalization indicated | Suicide attempted |

*Respiratory*

| **PARAMETER** | **GRADE 1 MILD** | **GRADE 2 MODERATE** | **GRADE 3 SEVERE** | **GRADE 4 POTENTIALLY LIFE-THREATENING** |
| --- | --- | --- | --- | --- |
| **Acute Bronchospasm** | Forced expiratory volume in 1 second or peak flow reduced to ≥ 70 to < 80% OR Mild symptoms with intervention not indicated | Forced expiratory volume in 1 second or peak flow 50 to < 70% OR Symptoms with intervention indicated OR Symptoms causing greater than minimal interference with usual social & functional activities | Forced expiratory volume in 1 second or peak flow 25 to < 50% OR Symptoms causing inability to perform usual social & functional activities | Forced expiratory volume in 1 second or peak flow < 25% OR Life-threatening respiratory or hemodynamic compromise OR Intubation |
| **Dyspnea or Respiratory Distress**  *Report only one* | Dyspnea on exertion with no or minimal interference with usual social & functional activities OR Wheezing OR Minimal increase in respiratory rate for age | Dyspnea on exertion causing greater than minimal interference with usual social & functional activities OR Nasal flaring OR Intercostal retractions OR Pulse oximetry 90 to < 95% | Dyspnea at rest causing inability to perform usual social & functional activities OR Pulse oximetry < 90% | Respiratory failure with ventilator support indicated (e.g., CPAP, BPAP, intubation) |

*Sensory*

| **PARAMETER** | **GRADE 1 MILD** | **GRADE 2 MODERATE** | **GRADE 3 SEVERE** | **GRADE 4 POTENTIALLY LIFE-THREATENING** |
| --- | --- | --- | --- | --- |
| **Hearing Loss** *≥ 12 years of age* | NA | Hearing aid or intervention not indicated | Hearing aid or intervention indicated | Profound bilateral hearing loss (> 80 dB at 2 kHz and above) OR Non-serviceable hearing (i.e., >50 dB audiogram and <50% speech discrimination) |
| *< 12 years of age (based on a 1, 2, 3, 4, 6 and 8 kHz audiogram)* | > 20 dB hearing loss at ≤ 4 kHz | > 20 dB hearing loss at > 4 kHz | > 20 dB hearing loss at ≥ 3 kHz in one ear with additional speech language related services indicated (where available) OR Hearing loss sufficient to indicate therapeutic intervention, including hearing aids | Audiologic indication for cochlear implant and additional speech-language related services indicated (where available) |
| **Tinnitus** | Symptoms causing no or minimal interference with usual social & functional activities with intervention not indicated | Symptoms causing greater than minimal interference with usual social & functional activities with intervention indicated | Symptoms causing inability to perform usual social & functional activities | NA |
| **Uveitis** | No symptoms AND Detectable on examination | Anterior uveitis with symptoms OR Medicamylasal intervention indicated | Posterior or pan-uveitis OR Operative intervention indicated | Disabling visual loss in affected eye(s) |
| **Vertigo** | Vertigo causing no or minimal interference with usual social & functional activities | Vertigo causing greater than minimal interference with usual social & functional activities | Vertigo causing inability to perform usual social & functional activities | Disabling vertigo causing inability to perform basic self-care functions |
| **Visual Changes** (assessed from baseline) | Visual changes causing no or minimal interference with usual social & functional activities | Visual changes causing greater than minimal interference with usual social & functional activities | Visual changes causing inability to perform usual social & functional activities | Disabling visual loss in affected eye(s) |

*Systemic*

| **PARAMETER** | **GRADE 1 MILD** | **GRADE 2 MODERATE** | **GRADE 3 SEVERE** | **GRADE 4 POTENTIALLY LIFE-THREATENING** |
| --- | --- | --- | --- | --- |
| **Acute Allergic Reaction** | Localized urticaria (wheals) with no medical intervention indicated | Localized urticaria with intervention indicated OR Mild angioedema with no intervention indicated | Generalized urticaria OR Angioedema with intervention indicated OR Symptoms of mild bronchospasm | Acute anaphylaxis OR Life-threatening bronchospasm OR Laryngeal edema |
| **Chills** | Symptoms causing no or minimal interference with usual social & functional activities | Symptoms causing greater than minimal interference with usual social & functional activities | Symptoms causing inability to perform usual social & functional activities | NA |
| **Cytokine Release Syndrome^9^** | Mild signs and symptoms AND Therapy (i.e., antibody infusion) interruption not indicated | Therapy (i.e., antibody infusion) interruption indicated AND Responds promptly to symptomatic treatment OR Prophylactic medications indicated for ≤ 24 hours | Prolonged severe signs and symptoms OR Recurrence of symptoms following initial improvement | Life-threatening consequences (e.g., requiring pressor or ventilator support) |
| **Fatigue or Malaise**  *Report only one* | Symptoms causing no or minimal interference with usual social & functional activities | Symptoms causing greater than minimal interference with usual social & functional activities | Symptoms causing inability to perform usual social & functional activities | Incapacitating symptoms of fatigue or malaise causing inability to perform basic self-care functions |
| **Fever** (non-axillary temperatures only) | 38.0 to < 38.6°C or 100.4 to < 101.5°F | ≥ 38.6 to < 39.3°C or ≥ 101.5 to < 102.7°F | ≥ 39.3 to < 40.0°C or ≥ 102.7 to < 104.0°F | ≥ 40.0°C or ≥ 104.0°F |
| **Pain^10^** (not associated with study agent injections and not specified elsewhere)  *Specify location* | Pain causing no or minimal interference with usual social & functional activities | Pain causing greater than minimal interference with usual social & functional activities | Pain causing inability to perform usual social & functional activities | Disabling pain causing inability to perform basic self-care functions OR Hospitalization indicated |
| **Serum Sickness^11^** | Mild signs and symptoms | Moderate signs and symptoms AND Intervention indicated (e.g., antihistamines) | Severe signs and symptoms AND Higher level intervention indicated (e.g., steroids or IV fluids) | Life-threatening consequences (e.g., requiring pressor or ventilator support) |
| **Underweight^12^ > 5 to 19 years of age** | NA | WHO BMI z-score < -2 to ≤ -3 | WHO BMI z-score < -3 | WHO BMI z-score < -3 with life-threatening consequences |
| **2 to 5 years of age** | NA | WHO Weight-for-height z-score  < -2 to ≤ -3 | WHO Weight-for-height z-score < -3 | WHO Weight-for-height z-score < -3 with life-threatening consequences |
| **< 2 years of age** | NA | WHO Weight-for-length z-score  < -2 to ≤ -3 | WHO Weight-for-length z-score < -3 | WHO Weight-for-length z-score < -3 with life-threatening consequences |
| **Weight Loss (excludes postpartum weight loss)** | NA | 5 to < 9% loss in body weight from baseline | ≥ 9 to < 20% loss in body weight from baseline | ≥ 20% loss in body weight from baseline OR Aggressive intervention indicated (e.g., tube feeding, total parenteral nutrition) |

9 Definition: A disorder characterized by nausea, headache, tachycardia, hypotension, rash, and/or shortness of breath.

10 For pain associated with injections or infusions, see the Site Reactions to Injections and Infusions section (page 23).

11 Definition: A disorder characterized by fever, arthralgia, myalgia, skin eruptions, lymphadenopathy, marked discomfort, and/or dyspnea.

12 WHO reference tables may be accesed by clicking the desired age range or by accessing the following URLs: <http://www.who.int/growthref/who2007_bmi_for_age/en/> for participants > 5 to 19 years of age and <http://www.who.int/childgrowth/standards/chart_catalogue/en/> for those ≤ 5 years of age.

*Urinary*

| **PARAMETER** | **GRADE 1 MILD** | **GRADE 2 MODERATE** | **GRADE 3 SEVERE** | **GRADE 4 POTENTIALLY LIFE-THREATENING** |
| --- | --- | --- | --- | --- |
| **Urinary Tract Obstruction** | NA | Signs or symptoms of urinary tract obstruction without hydronephrosis or renal dysfunction | Signs or symptoms of urinary tract obstruction with hydronephrosis or renal dysfunction | Obstruction causing life-threatening consequences |

*Site Reactions to Injections and Infusions*

| **PARAMETER** | **GRADE 1 MILD** | **GRADE 2 MODERATE** | **GRADE 3 SEVERE** | **GRADE 4 POTENTIALLY LIFE-THREATENING** |
| --- | --- | --- | --- | --- |
| **Injection Site Pain or Tenderness**  *Report only one* | Pain or tenderness causing no or minimal limitation of use of limb | Pain or tenderness causing greater than minimal limitation of use of limb | Pain or tenderness causing inability to perform usual social & functional activities | Pain or tenderness causing inability to perform basic self-care function OR Hospitalization indicated |
| **Injection Site Erythema or Redness^13^**  *Report only one*  *> 15 years of age* | 2.5 to < 5 cm in diameter OR 6.25 to < 25 cm2 surface area AND Symptoms causing no or minimal interference with usual social & functional activities | ≥ 5 to < 10 cm in diameter OR ≥ 25 to < 100 cm2 surface area OR Symptoms causing greater than minimal interference with usual social & functional activities | ≥ 10 cm in diameter OR ≥ 100 cm2 surface area OR Ulceration OR Secondary infection OR Phlebitis OR Sterile abscess OR Drainage OR Symptoms causing inability to perform usual social & functional activities | Potentially life-threatening consequences (e.g., abscess, exfoliative dermatitis, necrosis involving dermis or deeper tissue) |
| *≤ 15 years of age* | ≤ 2.5 cm in diameter | > 2.5 cm in diameter with < 50% surface area of the extremity segment involved (e.g., upper arm or thigh) | ≥ 50% surface area of the extremity segment involved (e.g., upper arm or thigh) OR Ulceration OR Secondary infection OR Phlebitis OR Sterile abscess OR Drainage | Potentially life-threatening consequences (e.g., abscess, exfoliative dermatitis, necrosis involving dermis or deeper tissue) |
| **Injection Site Induration or Swelling**  *Report only one*  *> 15 years of age* | Same as for **Injection Site Erythema or Redness,** *> 15 years of age* | Same as for **Injection Site Erythema or Redness,** *> 15 years of age* | Same as for **Injection Site Erythema or Redness,** *> 15 years of age* | Same as for **Injection Site Erythema or Redness,** *> 15 years of age* |
| ≤ *15 years of age* | Same as for **Injection Site Erythema or Redness,** *≤ 15 years of age* | Same as for **Injection Site Erythema or Redness,** *≤ 15 years of age* | Same as for **Injection Site Erythema or Redness,** *≤ 15 years of age* | Same as for **Injection Site Erythema or Redness,** *≤ 15 years of age* |
| **Injection Site Pruritus** | Itching localized to the injection site that is relieved spontaneously or in < 48 hours of treatment | Itching beyond the injection site that is not generalized OR Itching localized to the injection site requiring ≥ 48 hours treatment | Generalized itching causing inability to perform usual social & functional activities | NA |

13 Injection Site Erythema or Redness should be evaluated and graded using the greatest single diameter or measured surface area.

*Laboratory Values Chemistries*

| **PARAMETER** | **GRADE 1 MILD** | **GRADE 2 MODERATE** | **GRADE 3 SEVERE** | **GRADE 4 POTENTIALLY LIFE-THREATENING** |
| --- | --- | --- | --- | --- |
| **Acidosis** | NA | pH ≥ 7.3 to < LLN | pH < 7.3 without life-threatening consequences | pH < 7.3 with life-threatening consequences |
| **Albumin, Low** (g/dL; *g/L*) | 3.0 to < LLN *30 to < LLN* | ≥ 2.0 to < 3.0 *≥ 20 to < 30* | < 2.0 *< 20* | NA |
| **Alkaline Phosphatase, High** | 1.25 to < 2.5 x ULN | 2.5 to < 5.0 x ULN | 5.0 to < 10.0 x ULN | ≥ 10.0 x ULN |
| **Alkalosis** | NA | pH > ULN to ≤ 7.5 | pH > 7.5 without life-threatening consequences | pH > 7.5 with life-threatening consequences |
| **ALT or SGPT, High**  *Report only one* | 1.25 to < 2.5 x ULN | 2.5 to < 5.0 x ULN | 5.0 to < 10.0 x ULN | ≥ 10.0 x ULN |
| **Amylase (Pancreatic) or Amylase (Total), High**  *Report only one* | 1.1 to < 1.5 x ULN | 1.5 to < 3.0 x ULN | 3.0 to < 5.0 x ULN | ≥ 5.0 x ULN |
| **AST or SGOT, High**  *Report only one* | 1.25 to < 2.5 x ULN | 2.5 to < 5.0 x ULN | 5.0 to < 10.0 x ULN | ≥ 10.0 x ULN |
| **Bicarbonate, Low** (mEq/L; *mmol/L*) | 16.0 to < LLN *16.0 to < LLN* | 11.0 to < 16.0 *11.0 to < 16.0* | 8.0 to < 11.0 *8.0 to < 11.0* | < 8.0 *< 8.0* |
| **Bilirubin *Direct Bilirubin^14^, High***  *> 28 days of age* | NA | NA | > ULN | > ULN with life-threatening consequences (e.g., signs and symptoms of liver failure) |
| *≤ 28 days of age* | ULN to ≤ 1 mg/dL | > 1 to ≤ 1.5 mg/dL | > 1.5 to ≤ 2 mg/dL | > 2 mg/dL |
| ***Total Bilirubin, High***  *> 28 days of age* | 1.1 to < 1.6 x ULN | 1.6 to < 2.6 x ULN | 2.6 to < 5.0 x ULN | ≥ 5.0 x ULN |
| *≤ 28 days of age* | See Appendix A. Total Bilirubin for Term and Preterm Neonates | See Appendix A. Total Bilirubin for Term and Preterm Neonates | See Appendix A. Total Bilirubin for Term and Preterm Neonates | See Appendix A. Total Bilirubin for Term and Preterm Neonates |
| **Calcium, High** (mg/dL; *mmol/L*)  *≥ 7 days of age* | 10.6 to < 11.5 *2.65* to < *2.88* | 11.5 to < 12.5 *2.88* to < *3.13* | 12.5 to < 13.5 *3.13* to < *3.38* | ≥ 13.5 ≥ *3.38* |
| *< 7 days of age* | 11.5 to < 12.4 *2.88* to < *3.10* | 12.4 to < 12.9 *3.10* to < *3.23* | 12.9 to < 13.5 *3.23* to < *3.38* | ≥ 13.5 ≥ *3.38* |
| **Calcium (Ionized), High** *(mg/dL; mmol/L)* | > ULN to < 6.0 > ULN to < 1.5 | 6.0 to < 6.4 1.5 to < 1.6 | 6.4 to < 7.2 1.6 to < 1.8 | ≥ 7.2 ≥ 1.8 |
| **Calcium, Low** *(mg/dL; mmol/L)*  *≥ 7 days of age* | 7.8 to < 8.4 1.95 to < 2.10 | 7.0 to < 7.8 1.75 to < 1.95 | 6.1 to < 7.0 1.53 to < 1.75 | < 6.1 < 1.53 |
| *< 7 days of age* | 6.5 to < 7.5 1.63 to < 1.88 | 6.0 to < 6.5 1.50 to < 1.63 | 5.50 to < 6.0 1.38 to < 1.50 | < 5.50 < 1.38 |
| **Calcium (Ionized), Low** *(mg/dL; mmol/L)* | < LLN to 4.0 < LLN to 1.0 | 3.6 to < 4.0 0.9 to < 1.0 | 3.2 to < 3.6 0.8 to < 0.9 | < 3.2 < 0.8 |
| **Cardiac Troponin I, High** | NA | NA | NA | Levels consistent with myocardial infarction or unstable angina as defined by the local laboratory |
| **Creatine Kinase, High** | 3 to < 6 x ULN | 6 to < 10 x ULN | 10 to < 20 x ULN | ≥ 20 x ULN |
| **Creatinine, High** | 1.1 to 1.3 x ULN | > 1.3 to 1.8 x ULN OR Increase of > 0.3 mg/dL above baseline | > 1.8 to < 3.5 x ULN OR Increase of 1.5 to < 2.0 x above baseline | ≥ 3.5 x ULN OR  Increase of ≥ 2.0 x above baseline |
| **Creatinine Clearance^15^ or eGFR, Low**  *Report only one* | NA | < 90 to 60 ml/min or ml/min/1.73 m2 OR 10 to < 30% decrease from baseline | < 60 to 30 ml/min or ml/min/1.73 m2 OR ≥ 30 to < 50% decrease from baseline | < 30 ml/min or ml/min/1.73 m2 OR ≥ 50% decrease from baseline or dialysis needed |
| **Glucose**  *(mg/dL; mmol/L)*  *Fasting, High* | 110 to 125 6.11 to < 6.95 | > 125 to 250 6.95 to < 13.89 | > 250 to 500 13.89 to < 27.75 | > 500 ≥ 27.75 |
| *Nonfasting, High* | 116 to 160 6.44 to < 8.89 | > 160 to 250 8.89 to < 13.89 | > 250 to 500 13.89 to < 27.75 | > 500 ≥ 27.75 |
| **Glucose, Low** *(mg/dL; mmol/L)*  *≥ 1 month of age* | 55 to 64 3.05 to 3.55 | 40 to < 55 2.22 to < 3.05 | 30 to < 40 1.67 to < 2.22 | < 30 < 1.67 |
| *< 1 month of age* | 50 to 54 2.78 to 3.00 | 40 to < 50 2.22 to < 2.78 | 30 to < 40 1.67 to < 2.22 | < 30 < 1.67 |
| **Lactate, High** | ULN to < 2.0 x ULN without acidosis | ≥ 2.0 x ULN without acidosis | Increased lactate with pH < 7.3 without life-threatening consequences | Increased lactate with pH < 7.3 with life-threatening consequences |
| **Lipase, High** | 1.1 to < 1.5 x ULN | 1.5 to < 3.0 x ULN | 3.0 to < 5.0 x ULN | ≥ 5.0 x ULN |
| **Lipid Disorders**  (mg/dL; mmol/L)  **Cholesterol, Fasting, High**  ≥ 18 years of age | 200 to < 240 5.18 to < 6.19 | 240 to < 300 6.19 to < 7.77 | ≥ 300 ≥ 7.77 | NA |
| < 18 years of age | 170 to < 200 4.40 to < 5.15 | 200 to < 300 5.15 to < 7.77 | ≥ 300 ≥ 7.77 | NA |
| **LDL, Fasting, High**  ≥ 18 years of age | 130 to < 160 3.37 to < 4.12 | 160 to < 190 4.12 to < 4.90 | ≥ 190 ≥ 4.90 | NA |
| > 2 to < 18 years of age | 110 to < 130 2.85 to < 3.34 | 130 to < 190 3.34 to < 4.90 | ≥ 190 ≥ 4.90 | NA |
| **Triglycerides, Fasting, High** | 150 to 300 1.71 to 3.42 | >300 to 500 >3.42 to 5.7 | >500 to < 1,000 >5.7 to 11.4 | > 1,000 > 11.4 |
| **Magnesium^16^, Low** (mEq/L; mmol/L) | 1.2 to < 1.4 0.60 to < 0.70 | 0.9 to < 1.2 0.45 to < 0.60 | 0.6 to < 0.9 0.30 to < 0.45 | < 0.6 < 0.30 |
| **Phosphate, Low** (mg/dL; mmol/L)  > 14 years of age | 2.0 to < LLN 0.81 to < LLN | 1.4 to < 2.0 0.65 to < 0.81 | 1.0 to < 1.4 0.32 to < 0.65 | < 1.0 < 0.32 |
| 1 to 14 years of age | 3.0 to < 3.5 0.97 to < 1.13 | 2.5 to < 3.0 0.81 to < 0.97 | 1.5 to < 2.5 0.48 to < 0.81 | < 1.5 < 0.48 |
| < 1 year of age | 3.5 to < 4.5 1.13 to < 1.45 | 2.5 to < 3.5 0.81 to < 1.13 | 1.5 to < 2.5 0.48 to < 0.81 | < 1.5 < 0.48 |
| **Potassium, High** (mEq/L; mmol/L) | 5.6 to < 6.0 5.6 to < 6.0 | 6.0 to < 6.5 6.0 to < 6.5 | 6.5 to < 7.0 6.5 to < 7.0 | ≥ 7.0 ≥ 7.0 |
| **Potassium, Low** (mEq/L; mmol/L) | 3.0 to < 3.4 3.0 to < 3.4 | 2.5 to < 3.0 2.5 to < 3.0 | 2.0 to < 2.5 2.0 to < 2.5 | < 2.0 < 2.0 |
| **Sodium, High** (mEq/L; mmol/L) | 146 to < 150 146 to < 150 | 150 to < 154 150 to < 154 | 154 to < 160 154 to < 160 | ≥ 160 ≥ 160 |
| **Sodium, Low** (mEq/L; mmol/L) | 130 to < 135 130 to < 135 | 125 to < 130 125 to < 135 | 121 to < 125 121 to < 125 | ≤ 120 ≤ 120 |
| **Uric Acid, High** (mg/dL; mmol/L) | 7.5 to < 10.0 0.45 to < 0.59 | 10.0 to < 12.0 0.59 to < 0.71 | 12.0 to < 15.0 0.71 to < 0.89 | ≥ 15.0 ≥ 0.89 |

14 Direct bilirubin > 1.5 mg/dL in a participant < 28 days of age should be graded as grade 2, if < 10% of the total bilirubin.

15 Use the applicable formula (i.e., Cockroft-Gault in mL/min or Schwatrz in mL/min/1.73m2).

16 To convert a magnesium value from mg/dL to mmol/L, laboratories should multiply by 0.4114.

*Hematology*

| **PARAMETER** | **GRADE 1 MILD** | **GRADE 2 MODERATE** | **GRADE 3 SEVERE** | **GRADE 4 POTENTIALLY LIFE-THREATENING** |
| --- | --- | --- | --- | --- |
| **Absolute CD4+ Count, Low** (cell/mm3; *cells/L*)  *> 5 years of age (not HIV infected)* | 300 to < 400 *300 to < 400* | 200 to < 300 *200 to < 300* | 100 to < 200 *100 to < 200* | < 100 *< 100* |
| **Absolute Lymphocyte Count, Low**  (cell/mm3; *cells/L*)  *> 5 years of age*  *(not HIV infected)* | 600 to < 650 *0.600 x 10^9^ to < 0.650 x 10^9^* | 500 to < 600 *0.500 x 10^9^ to < 0.600 x 10^9^* | 350 to < 500 *0.350 x 10^9^ to < 0.500 x 10^9^* | < 350 *< 0.350 x 10^9^* |
| **Absolute Neutrophil Count (ANC), Low**  (cells/mm3; *cells/L*)  *> 7 days of age* | 800 to 1,000  *0.800 x 10^9^ to 1.000 x 10^9^* | 600 to 799  *0.600 x 10^9^ to 0.799 x 10^9^* | 400 to 599  *0.400 x 10^9^ to 0.599 x 10^9^* | < 400  *< 0.400 x 10^9^* |
| *2 to 7 days of age* | 1,250 to 1,500  *1.250 x 10^9^ to 1.500 x 10^9^* | 1,000 to 1,249  *1.000 x 10^9^ to 1.249 x 10^9^* | 750 to 999  *0.750 x 10^9^ to 0.999 x 10^9^* | < 750  *< 0.750 x 10^9^* |
| *≤ 1 day of age* | 4,000 to 5,000  *4.000 x 10^9^ to 5.000 x 10^9^* | 3,000 to 3,999  *3.000 x 10^9^ to 3.999 x 10^9^* | 1,500 to 2,999  *1.500 x 10^9^ to 2.999 x 10^9^* | < 1,500  *< 1.500 x 10^9^* |
| **Fibrinogen, Decreased** (mg/dL; *g/L*) | 100 to < 200 *1.00 to* < *2.00* OR 0.75 to < 1.00 x LLN | 75 to < 100 *0.75 to < 1.00* OR ≥ 0.50 to < 0.75 x LLN | 50 to < 75 *0.50 to < 0.75* OR 0.25 to < 0.50 x LLN | < 50 < *0.50* OR < 0.25 x LLN OR Associated with gross bleeding |
| **Hemoglobin^17^, Low** (g/dL; *mmol/L*)**^18^**  *≥ 13 years of age (male only)* | 10.0 to 10.9  *6.19 to 6.76* | 9.0 to < 10.0  *5.57 to < 6.19* | 7.0 to < 9.0  *4.34 to < 5.57* | < 7.0  *< 4.34* |
| *≥ 13 years of age (female only)* | 9.5 to 10.4  *5.88 to 6.48* | 8.5 to < 9.5  *5.25 to < 5.88* | 6.5 to < 8.5  *4.03 to < 5.25* | < 6.5  *< 4.03* |
| *57 days of age to < 13 years of age (male and female)* | 9.5 to 10.4  5.88 to 6.48 | 8.5 to < 9.5  5.25 to < 5.88 | 6.5 to < 8.5  4.03 to < 5.25 | < 6.5  < 4.03 |
| *36 to 56 days of age (male and female)* | 8.5 to 9.6  5.26 to 5.99 | 7.0 to < 8.5  4.32 to < 5.26 | 6.0 to < 7.0  3.72 to < 4.32 | < 6.0  < 3.72 |
| *22 to 35 days of age (male and female)* | 9.5 to 11.0  5.88 to 6.86 | 8.0 to < 9.5  4.94 to < 5.88 | 6.7 to < 8.0  4.15 to < 4.94 | < 6.7  < 4.15 |
| *8 to ≤ 21 days of age (male and female)* | 11.0 to 13.0  6.81 to 8.10 | 9.0 to < 11.0  5.57 to < 6.81 | 8.0 to < 9.0  4.96 to < 5.57 | < 8.0  < 4.96 |
| *≤ 7 days of age (male and female)* | 13.0 to 14.0  8.05 to 8.72 | 10.0 to < 13.0  6.19 to < 8.05 | 9.0 to < 10.0  5.59 to < 6.19 | < 9.0  < 5.59 |
| **INR, High**  *(not on anticoagulation therapy)* | 1.1 to < 1.5 x ULN | 1.5 to < 2.0 x ULN | 2.0 to < 3.0 x ULN | ≥ 3.0 x ULN |
| ***Methemoglobin (% hemoglobin)*** | 5.0 to < 10.0% | 10.0 to < 15.0% | 15.0 to < 20.0% | ≥ 20.0% |
| **PTT, High**  *(not on anticoagulation therapy)* | 1.1 to < 1.66 x ULN | 1.66 to < 2.33 x ULN | 2.33 to < 3.00 x ULN | ≥ 3.00 x ULN |
| **Platelets, Decreased** *(cells/mm3; cells/L)* | 100,000 to < 124,999 100.000 x 10^9^ to < 124.999 x 10^9^ | 50,000 to < 100,000 50.000 x 10^9^ to < 100.000 x 10^9^ | 25,000 to < 50,000 25.000 x 10^9^ to < 50.000 x 10^9^ | < 25,000 < 25.000 x 10^9^ |
| **PT, High**  *(not on anticoagulation therapy* | 1.1 to < 1.25 x ULN | 1.25 to < 1.50 x ULN | 1.50 to < 3.00 x ULN | ≥ 3.00 x ULN |
| **WBC, Decreased**  *(cells/mm3; cells/L)*  *> 7 days of age* | 2,000 to 2,499  2.000 x 10^9^ to 2.499 x 10^9^ | 1,500 to 1,999  1.500 x 10^9^ to 1.999 x 10^9^ | 1,000 to 1,499  1.000 x 10^9^ to 1.499 x 10^9^ | < 1,000  < 1.000 x 10^9^ |
| *≤ 7 days of age* | 5,500 to 6,999  5.500 x 10^9^ to 6.999 x 10^9^ | 4,000 to 5,499  4.000 x 10^9^ to 5.499 x 10^9^ | 2,500 to 3,999  2.500 x 10^9^ to 3.999 x 10^9^ | < 2,500  < 2.500 x 10^9^ |

17 Male and female sex are defined as sex at birth.

18 The conversion factor used to convert g/dL to mmol/L is 0.6206 and is the most commonly used conversion factor. For grading hemoglobin results obtained by an analytic method with a conversion factor other than 0.6206, the result must be converted to g/dL using the appropriate conversion factor for the particular laboratory.

*Urinalysis*

| **PARAMETER** | **GRADE 1 MILD** | **GRADE 2 MODERATE** | **GRADE 3 SEVERE** | **GRADE 4 POTENTIALLY LIFE-THREATENING** |
| --- | --- | --- | --- | --- |
| **Glycosuria (**random collection tested by dipstick) | Trace to 1+ or  ≤ 250 mg | 2+ or ˃ 250 to  ≤ 500 mg | > 2+ or > 500 mg | NA |
| **Hematuria** (not to be reported based on dipstick findings or on blood believed to be of menstrual origin) | 6 to < 10 RBCs per high power field | ≥ 10 RBCs per high power field | Gross, with or without clots OR With RBC casts OR Intervention indicated | Life-threatening consequences |
| **Proteinuria** (random collection tested by dipstick) | 1+ | 2+ | 3+ or higher | NA |
