# Supplementary material for: Association between low levels of HIV-1 DNA and HLA class I molecules in chronic HIV-1 infection
Source: PLoS One. 2022 Mar 15;17(3):e0265348. doi: 10.1371/journal.pone.0265348 (PMC8923435; doi:10.1371/journal.pone.0265348)
Supplement: Attachment — Submitted filename: Answers_Reviewers.docx [file pone.0265348.s004.docx]

Dear Editor,

We are very pleased that you have taken an interest in our work (Association between low levels of HIV-1 DNA and HLA class I molecules in chronic HIV-1 infection). We thank both reviewers for the insightful comments that helped to improve the manuscript.

We thank the reviewers for their positive comments and constructive suggestions. The raised concerns were carefully taken into consideration and addressed with appropriate changes in order to improve the manuscript. We answered to all comments and we hope that the revised manuscript will be accepted for publication.

Our revised submission contains changes to the manuscript.

Yours sincerely on behalf of all co-authors,

Camilla Muccini, MD, corresponding author

via Stamira D'Ancona 20

Milano, 20127, Italy

phone +390226437901

fax +390226437903

email: muccini.camilla@hsr.it

Reviewers' comments:

Reviewer's Responses to Questions

**Comments to the Author**

1. Is the manuscript technically sound, and do the data support the conclusions?

The manuscript must describe a technically sound piece of scientific research with data that supports the conclusions. Experiments must have been conducted rigorously, with appropriate controls, replication, and sample sizes. The conclusions must be drawn appropriately based on the data presented.

Reviewer #1: No

Reviewer #2: Yes

Reviewer #3: Yes

2. Has the statistical analysis been performed appropriately and rigorously?

Reviewer #1: No

Reviewer #2: Yes

Reviewer #3: Yes

3. Have the authors made all data underlying the findings in their manuscript fully available?

The [PLOS Data policy](http://www.plosone.org/static/policies.action#sharing) requires authors to make all data underlying the findings described in their manuscript fully available without restriction, with rare exception (please refer to the Data Availability Statement in the manuscript PDF file). The data should be provided as part of the manuscript or its supporting information, or deposited to a public repository. For example, in addition to summary statistics, the data points behind means, medians and variance measures should be available. If there are restrictions on publicly sharing data—e.g. participant privacy or use of data from a third party—those must be specified.

Reviewer #1: No

Reviewer #2: No

Reviewer #3: Yes

According to Plos Data policy we now provided the data supporting the findings described in our manuscript; data are provided in a .csv file (submitted as supporting information).

4. Is the manuscript presented in an intelligible fashion and written in standard English?

PLOS ONE does not copyedit accepted manuscripts, so the language in submitted articles must be clear, correct, and unambiguous. Any typographical or grammatical errors should be corrected at revision, so please note any specific errors here.

Reviewer #1: Yes

Reviewer #2: Yes

Reviewer #3: Yes

5. Review Comments to the Author

Please use the space provided to explain your answers to the questions above. You may also include additional comments for the author, including concerns about dual publication, research ethics, or publication ethics. (Please upload your review as an attachment if it exceeds 20,000 characters)

Reviewer #1: 1) The three reported signals are 21/78 vs. 1/18 (p=0.105), 1/78 vs. 3/16 (p=0.028), and 3/78 vs. 3/18 (p=0.087). If consider multiple testing adjustment, none is significant. Overall, there is no statistical power the declare any significant signal for the current study. More data or confirmation studies are needed.

The issue raised by the Reviewer is important and we agree on the fact that the study is not adequately powered to draw firm conclusions. However, not only fully-powered studies have been published in the scientific literature but also exploratory, proof-of-concept or pilot studies; despite these studies are characterized by a limited sample size, they have generally the merit to explore new scientific hypotheses.

We believe that the study design, the methods applied, the wording of our manuscript is in line with the small sample size; in fact, we proposed our work as an ***exploratory study***, we conducted univariate analyses only, we stated that HLA-B39 and -B55 ***might be associated*** with HIV-1 DNA <100 copies/10^6^PBMCs, we acknowledged among the limitations the small number of available patients and we concluded that the presence of HLA-B39 and -B55 ***may be associated*** with a lower level of total HIV-1 DNA, ***highlighting the need to deeply evaluate the role of HLA class I in affecting the size of reservoir.***

According to the Reviewer’s comment, we also included in the discussion two additional considerations on these issues.

Reviewer #2: This aimed at exploring the correlation between low levels of HIV-1 DNA and specific HLA alleles. Although results should be confirmed in a larger sample size, the study is interesting and clearly reported and discussed. However there are some minor points to be addressed:

- Please, report in more details the methodology used to assess the quantification of HIV-DNA and of residual viremia. This part can be also included in the supplementary material.

According to the Reviewer’s comment, we added more details in the method section on residual viremia quantification and we also included a specific reference for HIV-DNA quantification where the assay used for HIV-DNA is clearly explained (De Rossi A et al. Quantitative HIV-1 proviral DNA detection: a multicentre analysis. New Microbiol. 2010; 33:293-302).

- Please, specify if the assay allows to quantify total or integrated HIV-DNA.

We specified in the method that we quantified total HIV-DNA.

- Please, better specify the sentence “HIV-1 RNA was <50 copies/mL for a median time of 11.7 (10.6-14.0) years and absence of plasma residual viremia for 6.9 (6.2-7.2) years”. Does it mean that HIV-1 RNA or residual viremia was persistently undetectable or absent throughout the median time reported, with no episodes of viral blips?

Thanks for the comment; we added in the method section “without any viral blips” to better clarify the inclusion criteria of the study.

- Please, modify “Nadir CD4+ were” in “Nadir CD4+ T cell count was”

Thanks for the suggestion, we changed “Nadir CD4+ were” in “Nadir CD4+ T cell count was” in the text.

- Which was the selection criteria for the 88 patients screened for HLA?

Among 96 participants screened for HIV DNA, we have available HLA data for 88 patients.

- In the footnotes of Table 1, please report the statistical tests used to assess statistical significance.

According to the Reviewer’s comment, Table 1 now includes the statistical test applied to compare the two groups of patients.

- In Figure 1, please highlight those HLA alleles significantly associated with different levels of HIV-1 DNA.

We modified Figure 1 according to the Reviewer’s comment.

- Is there a correlation between HLA and the outcome of analytical treatment interruption?

We explored a possible correlation between HLA and the outcome of analytical treatment interruption but we did not find any association.

Reviewer #3: I suggest to better clarify some aspects:
- More information about study population should be useful: year of enrolment, type of regimens and line of ARV, if they ever failed… Moreover, since the discussion includes consideration about LTNP and elite controllers, it could be useful clarify if in this study population, this kind of patients are present or not (according to my understanding not)

According to the Reviewer’s comment, we added the requested information into the manuscript. The exploratory study here reported is a parent study of the APACHE trial that excluded LTNP and elite controllers (enrolled patients had to stop ART); so, we clarified this issue in the methods of the manuscript.

- I suggest to write in the main text the descriptive variables for all the patients, and to leave the differences among the two populations only in the table 1.

As suggested, we removed the overall column from the table and included in the text some overall patients’ characteristics.

- I suggest to clarify at which timepoint the HIVDNA test was performed: at time of enrolment in Apache study? It was repeated?

As reported in the manuscript, HIV-DNA was performed to screen participants of the APACHE study; it was repeated only in the patients included in the APACHE study who underwent analytic treatment interruption (ATI). HIV-DNA trend during ATI remained below 100 copies/10^6^PBMCs at all study visits except in two participants.

- The authors stated that HLA-B27 and B57 are well documented and associated to HIV outcomes. I suppose that their prevalence is described in Figure 1 (it is not readable, it is too small) but also a comment in the text should be added. E.g. if some associations has been found also with this two HLAs.

According to the Reviewer’s comment, we better clarified in the discussion section the role of HLA-B27 and HLA-B57 in our study (“In our analysis, these protective alleles were unexpectedly described with a low frequency among people with HIV-1 DNA <100 copies/106PBMCs and we did not find any correlation with the size of HIV-1 reservoir; further investigations on a larger sample size will be helpful to better interpret these findings.”)

Moreover, HLA-B27 was present in 5 participants (5.6%; 4 with HIV-DNA ≥100 copies/10^6^PBMCs and 1 with HIV-DNA <100 copies/10^6^PBMCs), while HLA-B57 in 4 (4.5%), all with HIV-DNA ≥100 copies/10^6^PBMCs.

- In the discussion, when HLA-B55 is mentioned, some previous data of other cohorts (references 17,18) seem to be in contrast with the findings of this study. Please give a comment on this.

Thanks for the suggestion, we made a comment in the discussion section about these data (“multicenter studies conducting on a greater number of participants should be designed to clarify the role of HLA-B55 in HIV-1 infection”).
