# Supplementary material for: Association between low levels of HIV-1 DNA and HLA class I molecules in chronic HIV-1 infection
Source: PLoS One. 2022 Mar 15;17(3):e0265348. doi: 10.1371/journal.pone.0265348 (PMC8923435; doi:10.1371/journal.pone.0265348)
Supplement: Attachment — Submitted filename: Reviewer Comments.docx [file pone.0265348.s003.docx]

The manuscript is will written and focuses on an interesting field.

I suggest to better clarify some aspects:

- More information about study population should be useful: year of enrolment, type of regimens and line of ARV, if they ever failed… Moreover, since the discussion includes consideration about LTNP and elite controllers, it could be useful clarify if in this study population, this kind of patients are present or not (according to my understanding not)
- I suggest to write in the main text the descriptive variables for all the patients, and to leave the differences among the two populations only in the table 1.
- I suggest to clarify at which timepoint the HIVDNA test was performed: at time of enrolment in Apache study? It was repeated?
- The authors stated that HLA-B27 and B57 are well documented and associated to HIV outcomes. I suppose that their prevalence is described in Figure 1 (it is not readable, it is too small) but also a comment in the text should be added. E.g. if some associations has been found also with this two HLAs.
- In the discussion, when HLA-B55 is mentioned, some previous data of other cohorts (references 17,18) seem to be in contrast with the findings of this study. Please give a comment on this.
